# The influence of arm swing constraint on thigh kinematics, heart rate response, and perceived exertion during the two-minute step test

**DOI:** 10.1371/journal.pone.0361590

**Published:** 2026-11-05

**Authors:** Gustavo de Oliveira Hoffmann, Thais Reichert, Matheus Henrique Esquelbeck, Mayra Alejandra Camacho Gama, Guilherme Parra Martini, Isabella Garcia Fernandes Lima, João Costa Rodrigues, Felipe Alves Marques, John Gerard Buckley, Roberta Castilhos Detanico Bohrer, André Luiz Felix Rodacki

**Affiliations:** 1 Universidade Federal do Paraná, Setor de Ciências Biológicas, Departamento de Educação Física, Rua Coronel Heráclito dos Santos, Jardim das Américas, Centro Politecnico, Curitiba, Paraná, Brazil; 2 Centro Universitário UniDomBosco, Departamento de Fisioterapia, Avenida Presidente Wenceslau Braz, Curitiba, Paraná, Brazil; 3 University of Bradford, Faculty of Engineering & Informatics, Bradford, England, United Kingdom; Pennsylvania State University Main Campus: The Pennsylvania State University - University Park Campus, UNITED STATES OF AMERICA

## Abstract

Holding a smartphone against the thigh provides a simple means of instrumenting the Two-Minute Step Test (2MST), but it constrains arm swing and may affect movement, cardiovascular responses, and perceived exertion. This study examined whether holding a smartphone against the thigh during the 2MST alters thigh kinematics, heart rate response, or perceived exertion in healthy young adults. Forty-five participants (27 men and 18 women; 19.5 ± 2.6 years) performed the 2MST under two counterbalanced conditions: unrestricted arm swing, with the smartphone secured to the right thigh using an elastic pouch, and constrained arm swing, with the smartphone held against the right thigh and the opposite hand placed on the contralateral thigh. Smartphone gyroscope data were used to determine step cycles, mean thigh peak angular velocity, cycle duration, variability, and the rate of change in thigh peak angular velocity. Heart rate response and perceived exertion were also recorded. Paired comparisons were performed using appropriate tests with Bonferroni correction. Step cycle counts were similar between constrained and unrestricted conditions (131 ± 20 vs 127 ± 19 cycles; p = 0.091). Mean thigh peak angular velocity, cycle duration, rate of change in thigh peak angular velocity, cycle duration variability, and heart rate response did not differ between conditions. Thigh peak angular velocity variability was higher in the constrained condition (p < 0.01; effect size = 0.68). Perceived exertion was also slightly higher in the constrained condition (12.6 ± 1.8 vs 11.9 ± 2.1; p = 0.024), but the difference was below the minimal detectable change threshold. These findings indicate that holding a smartphone against the thigh does not meaningfully alter overall 2MST performance, most thigh kinematic outcomes, or heart rate response, supporting this practical smartphone-based protocol.

## Introduction

Cardiorespiratory fitness is a key aspect related to the health of young and older adults with [1]and without [2] medical conditions. Measuring cardiorespiratory fitness has been generally assessed using standardized exercise treadmill or cycle ergometer tests, which are limiting factors to widespread measurements. As a treadmill or a cycle ergometer is not always readily available, an alternate assessment method is the 6-minute walk test [3], which has been used to assess cardiovascular fitness in young adults [4,5], and older adults [6,7]. However, such an assessment approach requires an uncluttered indoor corridor, a sports hall, or equivalent space, which may not always be available, which limits the use of such an assessment approach. A complementary cardiorespiratory test that can be conducted easily with limited space is the 2-minute step test (2MST). The 2MST was first presented in 1999 by Rikli and Jones [3] and requires the individual to march in place as fast as possible for 2 minutes, lifting the knees to a height corresponding to the center of the thigh segment on each step. The number of steps performed on the right side has been used as a criterion in a wide range of contexts, including physical fitness [4,5,8], functional capacity [9], frailty [10], sarcopenia [11], fall risk [12,13], and independence status [14]. In clinical scenarios, counting the step cycles completed is open to error and usually involves clinician time, whilst research determining step count (and other variables) using 3D motion capture requires specialist equipment. A simple alternative approach would be to hold a smartphone to the thigh when performing the 2MST, and then analysing the phone’s gyroscopic sensor data to determine thigh kinematic parameters to quantify step count and segmental kinematics [15].

Although in previous research, several aspects of the 2MST protocol are typically controlled (e.g., clear instructions, knee rising height, information to participants of the time elapsed since start, and the way steps must be performed – e.g., as fast as possible – [16]), there is little mention regarding the movement of the upper limbs while marching in place. Previous research on walking has shown that there are synchronous out-of-phase movements of the arms relative to the movements of the legs and that the amount of arm swing is linked with walking speed, as greater arm swing is expected when the speed is increased. The increased arm swing occurs to minimize the amount of trunk rotation induced by the increase in walking speed and helps keep the trunk motion smooth and non-jerky, which in turn helps maintain head stability [17]. There are other possible benefits to arm swing in walking, including it being linked with reducing the vertical displacement of the center of mass, the anterior-posterior angular momentum of the trunk, and the moment of the body about the vertical axis (e.g., the yaw moment) [18]. On the other hand, restricting arm movements during walking causes a notable increase from 8 to 34% in oxygen consumption and from 8 to 17% in heart rate compared to walking with normal arm swing [19]. It has also been demonstrated that there is an increased variability in the lower limb muscle recruitment when walking is performed with restricted arm movements [20]. It is unknown if constraining the arm swing during the 2MST changes segmental kinematic, cardiovascular, and perceptive parameters, as most studies were performed in walking (i.e., overground or treadmill). In stationary walking, the demands differ from those of forward walking, as there is no forward progression.

Recent work has demonstrated that the sensors embedded in smartphones (i.e., accelerometers and gyroscopes) can be used to quantify the kinematics of human movement (e.g.[15,21,22],). Using a smartphone in such a way to assess the 2MST would facilitate this test being used in a wide range of community and/or low-cost clinical settings, as well as for home/self-assessment scenarios. However, holding the smartphone to the thigh while marching in place will constrain the arm swing, which may impose different demands on movement control and/or the energetics than when the arms swing freely. The previous work deploying a smartphone to assess the 2MST attached the smartphone to the thigh using strapping, and thus the arms were free to swing with no constraints [15,21].

This study was designed to determine whether executing the 2MST with the arm swing being constrained by holding a smartphone to the thigh impacts thigh kinematics, heart rate response, or perceived exertion compared to executing the 2MST with the arms swinging freely. If holding the smartphone does not meaningfully alter these outcomes, this would support the use of a smartphone alone—without additional equipment such as straps or a phone pouch—to assess the 2MST. Our *a priori* hypotheses were that constraining arm swing by holding the smartphone against the thigh would not meaningfully alter: [1]test performance and thigh kinematic outcomes, including the number of step cycles, mean thigh peak angular velocity, cycle duration, rate of change in thigh peak angular velocity, and the cycle-to-cycle variability of peak angular velocity and cycle duration; or [2] physiological and perceptual responses, as indicated by heart rate response and perceived exertion.

## Methods

### Participants

Forty-five volunteers (18 females and 27 males; 19.5 ± 2.6 years, 1.7 ± 0.1 m, and 71 ± 12.6 kg) were recruited from the university’s local community and social projects and agreed to participate in the study. The eligibility criteria were: (a) age 18 years or older; (b) no recent history (within the last 6 months) of lower-limb injury or pain requiring the use of analgesics; and (c) no vestibular or neurological conditions that could affect test performance. In addition, individuals with prostheses or recent surgeries were excluded. All participants provided written informed consent, and the study was approved by the Ethics Committee of the Federal University of Paraná (approval number 7.412.815). Data were collected between August and November 2025.

An *a priori* sample size calculation was performed using G*Power software (version 3.1.9.7)* [23]. Because no previous study had directly evaluated the effect of restricting arm motion during the 2-min step test, a directly transferable effect-size estimate based on published data was not available. Therefore, a conventional moderate within-participant effect size (Cohen’s dz = 0.50) was used to determine the required sample size [24]. The calculation used a t-test for the difference between two dependent means (matched pairs), with a one-tailed hypothesis, α = 0.05, and statistical power of 0.95. These parameters resulted in a minimum required sample size of 45 participants (actual power = 0.951).

### Experimental design

Initially, participants received information about the experimental procedures, including a brief test demonstration, and performed a short familiarization marching-in-place trial (10–15 s) of both arm-swing conditions. They also received instructions on how to respond to the Rate of Perceived Exertion of 6–20 points (RPE scale) [25]. Then, a heart rate monitor (Polar Heart Rate Sensor, model H10) was attached to the participants’ chest (i.e., immediately below the pectoral muscles, with the area of the chest strap electrodes slightly moistened in water and directly applied to the bare chest). Then, they were requested to perform the 2MST in one of the arm-swing conditions – see below. The order of the two conditions was counterbalanced across participants by successively alternating the testing sequence. Participants were assigned alternately to the two possible testing sequences (A–B and B–A; A = unrestricted arm motion, B = restricted arm motion), resulting in 23 participants completing the testing sequence in one way and 22 completing it in the reverse sequence. Before the first experimental condition, resting heart rate was determined as the mean heart rate recorded during a 10-minute seated resting period. Following the first 2MST, participants remained seated for approximately five minutes, or longer, when necessary, until heart rate had returned to and stabilized around their individual resting mean. The second condition was initiated only after this recovery criterion had been met.

Participants performed a 2MST under two experimental conditions: with (SW_CON_ – constrained) and without constrained arm swing (SW_NORM_ – Unconstrained). During the 2MST, participants were instructed to march as fast as possible, touching an elastic band positioned immediately in front of them with the anterior surface of the distal end of their thighs (just superior to the femoral condyles). The height of the elastic band was adjusted to the height when standing of the midpoint between the iliac crest and the patella. In the SW_NORM_, a smartphone (iPhone 12, Apple Inc., model A2172, Cupertino, CA, USA) was positioned vertically against the anterior aspect of the upper third of the right thigh, with the screen facing the participant’s body, and secured using an adjustable pouch with elastic straps to minimize device displacement. For this condition, participants were allowed to swing their arms freely without additional instructions, and no control was applied. In the SW_CON,_ the phone was held by the participant’s right hand against the same region of the right thigh and maintained in the same vertical orientation, with the screen facing the participant’s body. The opposite hand was positioned over the upper third of the contralateral thigh at a corresponding height. This meant the swing of both arms was constrained in the SW_CON_ condition. Participants were instructed to maintain the smartphone in this position and minimize its displacement throughout the test. Fig 1 shows a schematic representation of a participant in both experimental conditions.

**Fig 1:**
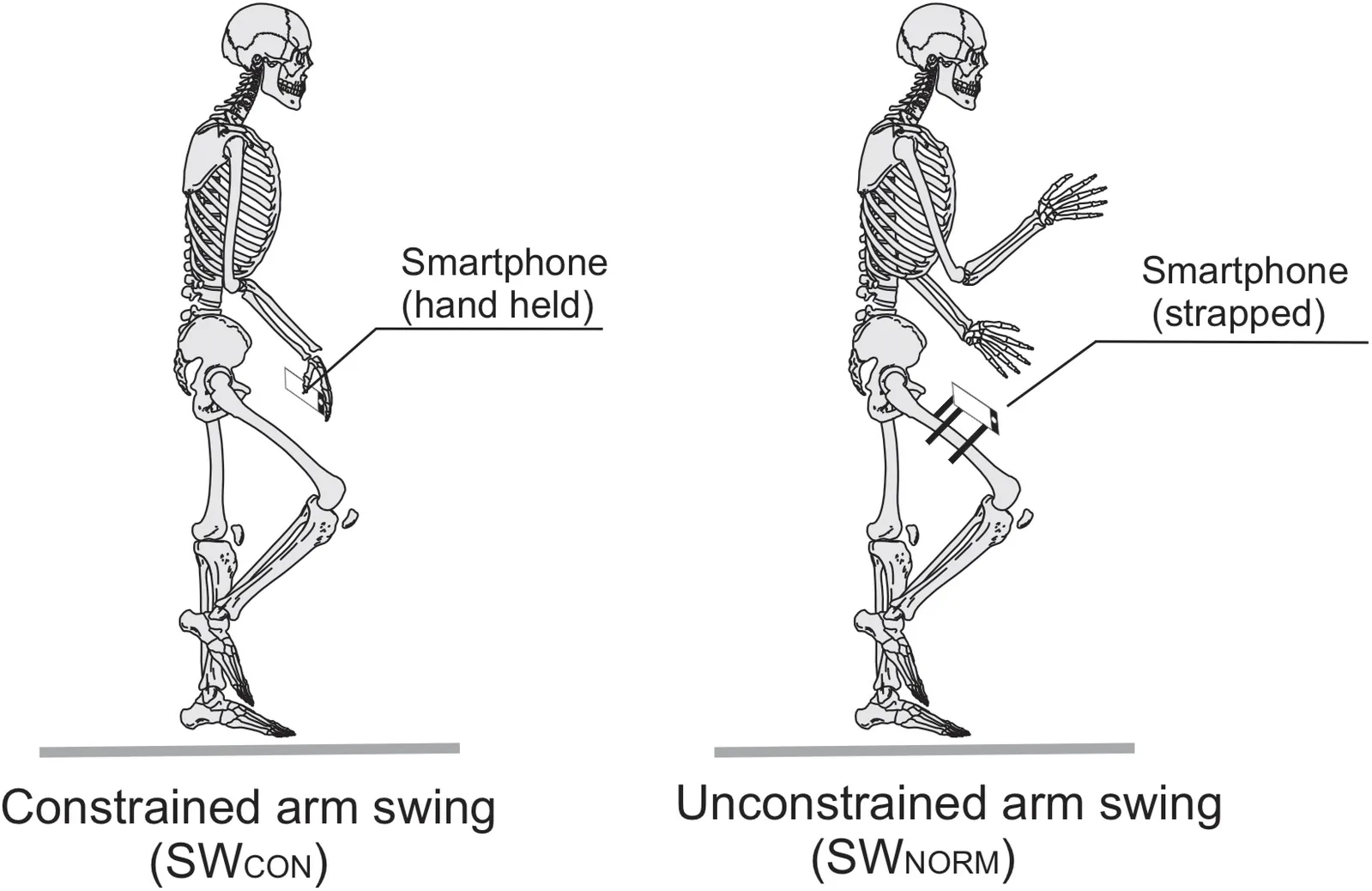
Schematic representation of a participant in the constrained arm swing (SW_CON_) and unconstrained arm swing (SW_NORM_) condition. Note: In the SW_CON_, the smartphone was secured by the participant’s hand (left image), while in the SW_NORM_, the smartphone was placed in an adjustable satchel with an elastic strap (right image).

Upon test completion of each condition, participants were asked to rate their perceived exertion using the RPE scale. No adverse symptoms (e.g., dizziness or claudication) were observed by participants during the tests.

### 2MST signal processing

For the analysis of the segmental kinematics, gyroscope data from the smartphone were extracted using commercial software (SensorLog, version 6.1) and subsequently processed and analyzed through a customized routine developed in Python (version 3.12.5; free and open-source software). The sampling frequency was initially standardized to 60 Hz (as the recorded signals vary between 58 and 62 Hz) using a cubic spline interpolation function [26]. Then, spatial and temporal variables of the right thigh were determined for each of the 11,385 step cycles using a machine learning–based calibration model.

Peak thigh angular velocity was estimated for each of the 11,385 step cycles using a previously developed and validated machine learning calibration model [27]. The model was trained using an independent dataset in which motion-capture-derived thigh peak angular velocity served as the supervised learning target. During model development, tri-axial smartphone gyroscope signals were filtered using a zero-lag, second-order low-pass Butterworth filter with a 6 Hz cutoff frequency. Time-domain predictors were extracted from fixed-length windows surrounding each peak and included peak values, selected percentiles, mean, standard deviation, root mean square, signal energy, high-amplitude width, number of zero crossings, cycle duration, and parabolically interpolated peak angular velocity from each gyroscope axis. The model consisted of a stacked regression framework combining ElasticNet and histogram-based gradient boosting regressors as base learners, with Ridge regression as the meta-learner. The resulting predictions were calibrated using isotonic regression, followed by an additional linear correction applied to the upper 5% of predicted values. In the independent validation dataset, the model presented a mean discrepancy of 1.0°·s^-1^ relative to motion capture, corresponding to approximately 0.3% of the mean reference value, with limits of agreement ranging from −15.4 to 17.5°·s^-1^. The previously trained model was applied to the present dataset without retraining. As the development and validation of the machine learning pipeline were not objectives of the present study, which focused on the effects of arm swing constraint, only the principal methodological steps are summarized here. Complete details regarding model development, training, validation, and performance are provided in [27].

The kinematic parameters analyzed in the present study included (a) the number of step cycles (STP – defined as the number of distinct positive peaks in the thigh peak angular velocity signal), (b) the mean thigh peak angular velocity (VEL – calculated as the mean peak thigh angular velocity for the repeated cycles), (c) and the mean cycle duration (DUR – the mean time between successive thigh angular velocity peaks over the two minutes). In addition, (d) the rate of change in thigh peak angular velocity (RCV) was determined for each participant (the slope of the line connecting the mean values from the initial and final 20s of the test). Finally, the coefficients of variability (across cycles) of the VEL and DUR were computed. The heart rate response was determined as the difference between the maximal heart rate obtained during the test and the estimated maximum age-predicted heart rate (FC_max_) (i.e., defined as 220 – participant’s age [28]).

### Statistics

Initially, all variables were analyzed using the Shapiro-Wilk and Levene tests to confirm data normality and homogeneity assumptions, respectively. The kinematic, cardiorespiratory, and perceptual parameters between the experimental conditions (SW_NORM_ vs. SW_CON_) were compared using a t-test for dependent measures. The Wilcoxon signed-rank test was applied when the normality and homogeneity assumptions were not met. The effect sizes were calculated. The family-wise error rate within each pre-specified family of outcomes was controlled. Specifically, the kinematic outcomes (m = 6; adjusted p = 0.0083), heart rate response outcomes (m = 2; adjusted p = 0.025), and perceptual outcomes (m = 1) were treated as separate families. The P-values were Bonferroni-adjusted a priori within each family (two-sided α = 0.05), except for the perceptual family (m = 1), in which the adjusted and raw p-values coincided. The minimal detectable change (MDC; [29]) was calculated for the variables that reached the significance levels. The statistical significance was set at p < 0.05, and all analyses were performed using the JASP software (Version 0.95.2).

## Results

A summary of the descriptive and inferential statistics for all between-condition comparisons is presented in Table 1. Participants performed a comparable number of step cycles in both experimental conditions (p = 0.091; ES = −0.35). No between-condition differences were observed for mean thigh peak angular velocity, mean cycle duration, rate of change in thigh peak angular velocity, or cycle-duration variability (all p ≥ 0.22; Table 1). Variability of thigh peak angular velocity was greater in SW_CON_ than in SW_NORM_ (p = 0.004; ES = 0.68). RPE was slightly higher in SW_CON_ than in SW_NORM_ (p = 0.024; ES = 0.24); however, the mean difference was below the MDC₉₅ of 3.78 a.u. Heart rate response was similar between conditions (p = 0.48; ES = −0.08; Table 1).

**Table 1:** Comparison of kinematic, perceptual, and heart rate outcomes between the constrained and unconstrained arm-swing conditions during the Two-Minute Step Test.

Outcome	SW_CON_	SW_NORM_	p-value	Effect size
Number of step cycles, cycles	131.4 ± 20.4	127.4 ± 18.6	0.091	−0.35
Mean thigh peak angular velocity, °·s^-1^	328.2 ± 23.2	325.7 ± 10.7	0.610	−0.10
Mean cycle duration, s	0.95 ± 0.17	0.97 ± 0.15	0.220	−0.18
Rate of change in peak angular velocity, °·s^-2^	−0.029 ± 0.169	−0.025 ± 0.097	0.900	−0.02
Variability of cycle duration, %	7.4 ± 13.6	6.6 ± 10.2	0.500	−0.12
Variability of peak angular velocity, %	15.3 ± 6.8	11.3 ± 4.5	0.004*	0.68
RPE, Borg 6–20 scale, a.u.	12.6 ± 1.8	11.9 ± 2.1	0.024*	0.24
Heart rate response, %FC_max_	66.5 ± 9.3	65.8 ± 9.0	0.480	−0.08

Data are presented as mean ± standard deviation. SW_CON_, constrained arm-swing condition; SW_NORM_, unconstrained arm-swing condition; RPE, rating of perceived exertion; FC_max_, age-predicted maximal heart rate.

## Discussion

This study aimed to determine whether constraining arm swing during the 2MST affects the thigh kinematics, heart rate response, and perceived exertion. The mean thigh kinematic and heart rate response parameters of the repeated stepping movements did not differ between conditions, indicating that arm swing does not play a relevant role in the 2MST outcomes. On the other hand, the perceived exertion showed a clinically non-significant change (see discussion below) between conditions, with participants perceiving the constrained condition slightly harder than when their arms were allowed to swing freely.

The number of steps (i.e., right limb cycles) observed in the present study (~130 cycles) was substantially higher than in many reports in the literature [7,16]. This discrepancy likely reflects sample characteristics, as many prior studies focused on older adults or individuals with clinical conditions, who typically complete fewer cycles compared to young healthy participants, which was the type of participants in the present study. The group mean number of cycles completed in the present study is equivalent to investigations with participants of similar age (e.g., ~ 25 years; ~ 123 cycles; [8]).

The present findings diverge from prior studies that restricted arm swing during treadmill [30] or overground [31] walking because the mechanics of marching in place differs fundamentally from forward gait. In unrestricted walking, forward progression of the center of mass (CoM) is generated by a clear propulsive anteroposterior push-off of the stance limb while the contralateral limb advances [32]. As the CoM moves forward, the pelvis typically rotates toward the swing limb, carrying the hip joint center forward and lengthening step length—the so-called “pelvic step” [33]. This transverse pelvic rotation tends to increase whole-body yaw (longitudinal) angular momentum, which is counteracted by contralateral arm swing; the arms provide a counter-rotation that helps balance whole-body angular momentum and reduce lateral CoM displacement, yielding smoother, less jerky motion of the CoM [34]. By contrast, marching in place has no forward progression, so the transverse “pelvic step” that aids step length and CoM smoothing in overground gait is minimal. When stepping in place, the CoM will shift predominantly in the mediolateral direction. To initiate the movement and lift the leg, the intended swing limb is unloaded, shifting the CoM toward the stance limb [32]. After reaching the maximal excursion during swing, the swing foot is placed back adjacent to the stance foot, keeping overall CoM excursion within minimal limits in the anterior-posterior direction. Given this geometry, arm swing is less likely to play a major role in controlling pelvic motions in the transverse (pelvic rotation) or frontal (pelvic list) planes during marching in place, as the yaw angular momentum is considerably reduced. Consistent with this, constraining arm swing (e.g., by securing a smartphone against the thigh with the hand, which limits arm motion) produced thigh kinematics similar to those observed when participants’ arms swung freely.

The results also indicate that although cycle duration variability was similar across conditions, the variability in mean thigh peak angular velocity was greater in the constrained condition compared with the unconstrained condition. This finding agrees with previous reports indicating that constraining arm swing causes greater variability while walking on a treadmill or overground [31,35]. One possible explanation for the greater variability in thigh angular velocity in the constrained condition may relate to changes in angular momentum associated with restricting arm swing. During walking, contralateral arm swing counteracts angular momentum generated by the lower limbs, reducing net whole-body angular momentum about the vertical axis [18]. Thus, constraining arm swing could have affected this process, and the unfamiliarity of this constraint could have contributed to the increased variability in peak thigh angular velocity. Another possible explanation is that requiring participants to hold the smartphone themselves increased exertion, which led to greater variability. In support of this, it is notable that participants’ rating of perceived exertion (RPE) was higher in the constrained condition compared with the unconstrained condition. It is also possible that the act of holding the phone to the thigh increased variability in peak thigh angular velocity by creating greater relative motion between the thigh and the phone (e.g., greater and more variable soft tissue motion) compared with when the phone was strapped to the thigh via the elastic pouch*.*

Although the restriction of arm movement did not result in an HR increase, a RPE elevation of approximately 0.7 a.u. was observed. However, such a difference was far below the minimum detectable change (MDC₉₅ = 3.78 a.u.) and presented a small effect size, indicating that such a variation may likely be attributable to measurement error or intra-individual variations rather than an actual change in perceived exertion. The slight increase in RPE for the constrained condition may be due to the trend increase (p = 0.09) in the number of steps completed for the constrained compared to the arms-free condition (which on average was around 4 more steps). It is also plausible that the slightly increased RPE may be related to the greater motor and postural control effort required to compensate for the angular momentum loss caused by the task constraint [31]. The increase in central command to stabilize the trunk and pelvis may heighten the conscious perception of effort, even in the absence of a significant cardiorespiratory response [36]. It is also plausible that arm swing constraints may have constituted a novelty that participants were not used to performing in their daily routines. Thus, discrete RPE fluctuations may reflect an additional neural and postural demand of a novel and unusual task rather than a global physiological overload.

Observing a similar HR in the 2MST with and without arm swing, contrasts with previous findings that reported a greater HR response (8–17%) when arm swing was constrained during walking in young men [19]. In that study, oxygen consumption and energy expenditure increased significantly during constrained arm swing, corroborating other findings in this area [37]. Although there is no consensus regarding why cardiorespiratory responses and energy cost are greater during walking with constrained arm swing, it has been suggested that arm swing causes a reduced vertical excursion of the CoM [34,38]. During forward walking, arm swing also counteracts the angular momentum generated by pelvic and trunk rotation and may reduce the muscular effort required to stabilize the trunk and control CoM motion. Restricting arm swing may therefore require greater compensatory activation of the trunk and lower-limb muscles, increasing metabolic demand and heart rate. In contrast, marching in place involves no forward progression, limited anteroposterior CoM displacement, and likely less transverse pelvic rotation and whole-body yaw angular momentum. Because body weight is transferred predominantly in the mediolateral direction, the arms may provide less mechanical counterbalance than during forward walking. Consequently, constraining arm swing during the 2MST may require little additional stabilizing work, which could explain the similar heart rate responses between conditions. However, this mechanism remains speculative because CoM motion, muscle activation, and oxygen consumption were not directly assessed. Within the present sample of young healthy adults, constraining arm swing did not increase the cardiovascular demand of the 2MST. Although perceived exertion was slightly higher in the constrained condition, the magnitude of this difference remained below the minimal detectable change, indicating that it was unlikely to represent a meaningful perceptual alteration.

From a methodological and implementation perspective, using the smartphone’s inertial sensors for analysing the 2MST offers significant practical and scientific benefits. Regarding applicability, it is an affordable, widely available, and universal solution that reduces logistical challenges and boosts the scalability of field measurements. A specific advantage of the proposed protocol is that holding the smartphone with the participant’s bare hands, which constrains arm swing, produced comparable outcomes to when arm swing was permitted. Therefore, the 2MST can be analysed without additional apparatuses (e.g., belts, armbands, pouches, or mounts). This can decrease setup time, reduce costs, improve procedural compliance, increase standardization, and simplify instructions. If a smartphone App is developed to provide 2MST outcomes automatically (from analysis of the phone’s gyroscopic sensor), this would facilitate using the 2MST to assess functional capacity in a wide range of low-cost community and clinical settings as well as for home/self-assessment scenarios.

## Limitations

During both conditions, ratings of perceived exertion (RPE; Borg 6–20 scale) were recorded at the end of each 2MST; however, participants did not undergo a comprehensive familiarization period. This likely increased within-subject measurement error. In particular, without prior anchoring and repeated exposures, individuals tend to rely on internal cues that are often inconsistent (e.g., local muscular discomfort, coordination novelty, or attentional load) rather than a stable global sense of effort/exertion. This concern is pertinent for the non–arm-swing condition (NSW), since constraining the arms and holding the smartphone may introduce changes in the thigh kinematics, heart rate, or RPE responses.

Other studies exploring oxygen consumption (e.g., VO₂) or electromyographic measurements could explore the effects of arm swing restriction deeper and confirm our suppositions. This study used indirect markers (HR and RPE), which are useful in field contexts but less sensitive for detecting subtle differences in metabolic cost between conditions. Future studies should integrate a portable metabolic analyzer synchronized with smartphones to estimate several parameters.

## Conclusions

The *a priori* hypotheses were largely supported. Constraining arm swing by holding a smartphone against the thigh did not alter the number of step cycles, mean thigh peak angular velocity, cycle duration, rate of change in thigh peak angular velocity, cycle-duration variability, or heart rate response. However, thigh peak angular velocity variability was higher in the constrained condition, and perceived exertion was slightly elevated, although the latter difference remained below the minimal detectable change threshold. Taken together, these findings suggest that holding a smartphone against the thigh can provide an expedient and pragmatic means to evaluate the 2MST as the approach doesn’t substantially affect the main outcomes of the 2MST in healthy young adults, and thus they also provide support for the feasibility of smartphone-based assessments without additional attachment equipment. However, given that holding the phone to the thigh did affect the variability in the thigh angular velocity some caution may be needed in using the highlighted approach in all scenarios. This approach may reduce setup time and logistical requirements, thereby improving the scalability of field-based, clinical, and potentially home-based assessments.

## Supporting information

S1 File Ethics Approval form: Part a) English translated version and Part b) Original Portuguese version. (PDF)

S2 File PLOS questionnaire on inclusivity in global research. Completed questionnaire on inclusivity in global research, as requested by PLOS ONE.
(DOCX)

S3 File Participant-level dataset underlying the findings reported in this study. The dataset contains the demographic characteristics and the participant-level kinematic, heart rate, and rating of perceived exertion outcomes obtained under the constrained arm-swing condition (SW_CON_) and the unconstrained arm-swing condition (SW_NORM_).
(XLSX)
