# Supplementary material for: The influence of arm swing constraint on thigh kinematics, heart rate response, and perceived exertion during the two-minute step test
Source: PLoS One. 2026 Nov 5;21(11):e0361590. doi: 10.1371/journal.pone.0361590 (PMC13739110; doi:10.1371/journal.pone.0361590)
Supplement: Supplementary file 1 [file pone.0361590.s001.pdf]

RESEARCH PROJECT INFORMATION

Research Title:
Assistive Technology for Integrated Clinical-Functional Assessment and Exercise Prescription for Older Adults Using the Brazilian Public Health System (SUS)

Principal Investigator:
André Luiz Felix Rodacki

Thematic Area:

Version: 2

CAAE:
83996424.4.0000.0102

Proposing Institution:
Department of Physical Education – Federal University of Paraná (UFPR)

Primary Sponsor:
Funding Authority for Studies and Projects (FINEP)

ETHICS COMMITTEE OPINION INFORMATION

Opinion Number: 7,310,210

Project Presentation

This opinion was prepared based on the information available in Plataforma Brasil.

The project entitled **"Assistive Technology for Integrated Clinical-Functional Assessment and Exercise Prescription for Older Adults Using the Brazilian Public Health System (SUS)"**, coordinated by Professor André Luiz Felix Rodacki from the Department of Physical Education at UFPR, is being conducted in collaboration with Professors Alessandro Brawerman, Dieval Guiselini, Gleber Pereira, José Luiz Padilha da Silva, Luciana Puchalski Kalinke, Natália Boneti Moreira, Paulo Cesar Barauce Bento, and Roberta Castilhos Detanico Bohrer.

This project was approved by FINEP under the public call **"MCTI/FINEP/CROSS-CUTTING ACTION – ASSISTIVE TECHNOLOGY" (Reference Number 2137/22)**, which aimed to support institutional projects for the development of the National System of Assistive Technology Laboratories (SisAssistiva-MCTI). Approval of the proposal was associated with funding of **R$ 3,903,657.79** (three million, nine hundred and three thousand, six hundred and fifty-seven reais and seventy-nine centavos). The research budget is **R$ 8,400.00**.

The study will be conducted from **November 2024 to December 2027**. The study population described in the cover sheet consists of **10,000 older adults aged between 60 and 80 years**.

The study is composed of several phases, including: a) **Validation of tests** (adaptation of clinical tests and questionnaires) for implementation in a smartphone-based electronic environment; b) **Diagnostic assessment of older adults** c) **Assessment of older adults attending Primary Health Care Units (UBSs)** through an application specifically developed for this purpose; and d) **Provision of an intervention program specifically designed for older adults**, delivered through smartphones.

The research site for **Phase 1** of the study will be the **Center for Motor Behavior Studies (CECOM)**, located within the Department of Physical Education, Biological Sciences Sector, at the Polytechnic Center of the Federal University of Paraná (UFPR). Subsequent phases, specifically **Phases 2 and 4** of the study (Pilot Study and Training), will be conducted in the Health Care Units designated by the municipalities participating in this project.

According to the investigators, the first phase, referred to as the **validation phase**, aims to determine whether measurements obtained from smartphone sensors (accelerometers and gyroscopes) are comparable to those obtained from equipment considered the gold standard. Approximately **200 participants** are expected to be sufficient for this phase.

Once valid and representative measurements from a set of clinical tests can be reliably collected using smartphones, the algorithms will be integrated into an application designed to assist primary health care professionals in administering questionnaires and conducting physical tests.

After the application becomes available, a **pilot study** will be conducted to evaluate its ability to obtain measurements from older adults in a limited number of Primary Health Care Units. To ensure the reliability of the measurements, health professionals working in these units will receive training on operating the application. Approximately **8,000 older adults** are expected to be assessed during this phase.

Once the issues and challenges identified during the pilot phase have been resolved, a **large-scale data collection process** will be conducted across all participating Primary Health Care Units. The resulting data will be used in Artificial Intelligence processes to determine whether questionnaires and physical tests can assist in diagnosing the study's primary outcomes.

If successful, the resulting database may be used to identify individuals with a greater predisposition (increased risk) to the study outcomes. This information will enable Municipal Health Departments to implement appropriate preventive actions.

The methods proposed for assessing older adults are widely used and well established in the scientific literature.

Finally, the last phase of the project involves providing physical activity programs specifically designed for older adults, delivered via smartphones. The physical activity program is particularly relevant because it will enable sedentary older adults to increase their physical activity levels and improve their health indicators in an attractive, free-of-charge manner, with minimal physical resources required (e.g., chairs, towels, PET bottles). In addition, older adults may benefit from such programs because participation does not require transportation, as all

activities can be performed at home. The training program will follow previously published and well-established guidelines.

Among the expected outcomes, the project aims to provide a low-cost physical activity program that is effective in increasing physical activity levels among older adults and improving their health status according to the proposed diagnostic measures. The collected data will be subjected to analyses involving Artificial Intelligence for the prediction of health conditions, including frailty, fatigue, mobility limitations, and falls. Therefore, the potential benefits to both Science and Society are evident.

Furthermore, participants will have rapid and accessible access to the results of their assessments through their smartphones, allowing their performance to be interpreted using reference values established by the study. In addition, the data will be stored in such a way that participants (and the professionals responsible for their clinical follow-up) will be able to monitor their health status over time. Finally, participants will have the opportunity to benefit from the physical activity programs offered through the application, which will be provided free of charge.

Inclusion Criteria

For the validation of the functional tests (Phase 1 of the study), specific inclusion and exclusion criteria will be applied. Individuals aged over 60 years (between 60 and 80 years of age), of either sex, who voluntarily agree to participate in the study will be eligible for inclusion. For the subsequent phases of the study, individuals aged 60 years or older (between 60 and 80 years of age), of either sex, who voluntarily agree to participate will be included.

There are no restrictions regarding participation in any of the tests associated with the project, as all assessments are easy to perform and constitute standard clinical practice in geriatrics. In general, there are also no restrictions related to different health conditions, since the proposed tests are routinely applied to individuals with such diagnoses.

Exclusion Criteria

Individuals presenting severe cognitive or neurological impairments that prevent them from performing the functional tests, completing the questionnaires, or understanding the instructions will be excluded from the study. All eligibility criteria will be identified through the initial screening conducted by the researcher. Participants who have not signed the Free and Informed Consent Form specific to this phase of the study (ICF 1) will not be included. If they have not signed the corresponding Informed Consent Forms, they will also not be included in the subsequent phases of the study. Participants who have not signed the Free and Informed Consent Forms specifically developed for the subsequent phases of the study (ICF 2 and ICF 3) will not be included.

Research Objectives

Primary Objective

The proposal aims to establish a standardized and integrated clinical-functional assessment system (functionality, perceptual-cognitive factors, and fall risk) across different health services using measurements obtained through smartphone sensors. In addition, it seeks to determine the effectiveness of a home-based exercise program delivered through assistive technology for older adults receiving care through the Brazilian Public Health System (SUS).

Secondary Objectives

1. To implement questionnaires within the system/application to assess fall history, cognitive decline, frailty status, fear of falling, fatigue status, physical activity levels, and sedentary behavior;
2. To develop and validate smartphone-based physical-functional performance testing algorithms capable of identifying reduced mobility and fall risk;
3. To identify potential diagnostic and monitoring relationships between health status and disease development based on sociodemographic information and clinical and physical-functional assessments;
4. To understand health-state transitions during aging through the longitudinal assessment history of participants;
5. To establish reference values for clinical physical-functional tests according to different age groups and health-related characteristics;
6. To identify fatigue levels among older cancer survivors;
7. To propose a remotely monitored physical activity program delivered through smartphones;
8. To evaluate the quality of life of older cancer survivors during participation in the remotely monitored physical activity program;
9. To analyze the effectiveness of a remotely monitored home-based exercise program delivered through smartphones on mobility and perceptual-cognitive outcomes in older adults.

Risk and Benefit Assessment

Risks

When answering the questionnaires, participants may experience some degree of discomfort or embarrassment due to questions of a personal nature, such as age, educational level, and number of diagnosed diseases. However, such questions are standard in virtually all research studies and are also routinely collected when participants are enrolled in intervention programs.

During the performance of the physical tests, participants may experience very mild fatigue and, in some cases, mild delayed-onset muscle soreness. Fatigue and delayed-onset muscle soreness are normal physiological responses and are expected to disappear quickly.

For participants enrolled in the home-based exercise program, it is possible that fatigue and muscle or joint discomfort may occur during and/or after exercise sessions due to the effort inherent to physical activity, particularly among individuals who are not accustomed to regular exercise. Participants may also experience episodes of imbalance or even falls if they do not follow the recommended safety and prevention guidelines. Safety instructions will be provided at the beginning of each session through the application, emphasizing the use of safe and stable materials (e.g., chairs) during the activities. Although such risks are inherent to virtually all assessment procedures and exercise programs, adverse events among participants are rare. It should also be emphasized that health care professionals working in Primary Health Care Units (UBSs) will receive training to administer the tests. Furthermore, all proposed tests are clinically safe and have been applied extensively among older adults both nationally and internationally.

Benefits

Based on the study findings, participants will receive, as a direct individual benefit, information regarding their physical-functional capacity through the application, with results interpreted according to age-specific reference values. These benefits may provide participants with a better understanding of their ability to perform functional activities. This information may encourage them to adopt a more active lifestyle and to engage in regular physical exercise. For older adults participating in the home-based exercise program, direct individual benefits are expected, including improved functionality, which may, in turn, enhance their ability to perform activities of daily living and reduce the risk of falls. Indirectly, this study may have a positive impact on the scientific community by demonstrating that clinical-functional assessments performed using assistive technologies (smartphones) can replace expensive equipment that is often inaccessible to the broader user community of the Brazilian Public Health System (SUS). Additionally, the implementation of remotely monitored, home-based exercise programs delivered via smartphones provides greater reach and accessibility, allowing individuals to exercise at home at any time without the need for transportation or reliance on others to travel to specific exercise facilities. These features represent important health promotion and disease prevention strategies for the growing older adult population. This study is expected to provide diagnostic tools for public health services. Advances in these tools include adapting several questionnaires to electronic formats and establishing local reference values, which are currently unavailable. Furthermore, the storage of assessment data will enable longitudinal monitoring of participants and facilitate the observation of transitions in health states, including frailty, fatigue, mobility, and other related outcomes. In addition, the study is expected to enhance the technological tools currently available, potentially allowing the widespread implementation of several clinical tests and the establishment of normative values for the community. Likewise, it is anticipated that public health services will be provided with accurate, objective, and cost-effective methods for measuring performance in a variety of physical tests, particularly because equipment considered the gold standard (e.g., force platforms) is generally inaccessible to professionals working in primary health care settings. Among the expected outcomes, the project also aims to provide a low-cost physical activity program capable of effectively increasing physical activity levels among older adults and improving their health status according to the proposed diagnostic measures. The study data may provide valuable evidence to support public health administrators in improving or revising health policies, ensuring that decisions are based on objectively measured parameters. The findings may also serve as a reference for other municipalities seeking

to adopt comparable monitoring and intervention strategies. Furthermore, the study is expected to contribute to the publication of scientific and educational materials, including journal articles, book chapters, books, and instructional resources, while also supporting the training of better-qualified professionals involved in health care processes for older adults who use services provided by Primary Health Care Units.

Comments and Considerations Regarding the Research

The study is well designed and has substantial social impact. In addition to the direct benefits provided to participants, its indirect benefits are particularly noteworthy. This is an intervention study aimed at developing diagnostic tools for public health services and physical activity programs. This may also provide significant benefits to health care administrators and decision-makers by expanding access to assessments and enabling the longitudinal monitoring of the health status of the community served by the Brazilian Public Health System (SUS). The study also aims to provide a physical activity program that can be implemented at low cost and is effective in increasing physical activity levels among older adults while improving their health status according to the proposed diagnostic measures.

Considerations Regarding the Required Submission Documents

The required documents were submitted in response to the previously identified pending issues.

Recommendations None.

Conclusions, Pending Issues, and List of Inadequacies

Considering that the explanations provided by the investigators complement the ethical review of the study and confirm the researchers' commitment to conducting the study in accordance with ethical principles, the justifications submitted are reproduced below.

1. Required Submission Documents - Committee Comment: The study identifies the Municipal Health Departments of Curitiba and the surrounding Metropolitan Region as research sites. However, letters of institutional agreement were submitted only from the Curitiba Municipal Health Department (SMS Curitiba), and the remaining municipal health departments were not identified as participating institutions. Clarification was requested. **Response:** The Committee's assessment is correct. However, the current transition of municipal administrations and municipal health department leadership has prevented the involved municipalities from formally issuing statements of adherence to the proposal, as such participation depends upon local approval. At present, we are awaiting review of the proposal by the new municipal administrations (2025) and confirmation of the intentions expressed during discussions held throughout 2024. Accordingly, amendments including letters of agreement from municipalities that formally join the project will be submitted throughout 2025. **2. Regarding the Cover**

Sheet - Committee Comment: The cover sheet indicates a sample of 10,000 participants. However, Section 6.1.4 ("Training of Primary Health Care Unit Professionals") states that during this phase (the first year of implementation), at least 10% of older adults from Curitiba and the Metropolitan Region are expected to be assessed (approximately 18,000 older adults), with these numbers increasing in subsequent years. Clarification regarding the sample size was requested and, if necessary, revision of the cover sheet. **Response:** The reviewer is correct. The number of participants was adjusted based on the estimated assessment of approximately **18,000 older adults** residing in the municipality of Curitiba and its Metropolitan Region. An updated cover sheet was submitted accordingly (File: *Folha_rosto_ass_05_12.pdf*). **3. Concerning the Use of Images Committee Comment:** The project states that, during testing, participants will hold a smartphone at the level of the sternum (center of the chest) with both hands and, from a seated position, will initiate the test. Simultaneously, a video camera will be positioned perpendicular to the sagittal plane of movement, focused at shoulder height. The Committee requested the inclusion of an Image and/or Voice and/or Medical Record Use Authorization Form, in accordance with Model 15 available on the CEP/SD website. **Response:** We clarify that the images recorded in the sagittal plane are intended solely to provide the coordinates of body landmarks, which will be used to calculate movement parameters. Therefore, no images in which participants can be identified will be disseminated outside the context necessary for data analysis. The videos will be used exclusively for internal data processing purposes and will remain in a restricted-access environment. Nevertheless, in order to address the Committee's concerns, an Image Use Authorization Form was added as an additional document, as suggested (File: *Termo_de_imagem.docx*). **4. Concerning Informed Consent Form 1 (ICF 1) Committee Comment:** The consent form states: *"The expenses necessary for participation in the study (assessments, disposable materials, etc.) are not your responsibility, and you will not receive any financial compensation for participating in the study. If necessary, transportation expenses incurred by participants traveling to the Department of Physical Education at the Polytechnic Campus of UFPR will be reimbursed through public transportation fare cards."*(Resolution 466/2012, Item II.21 and Item IV.3, subsection g). The Committee requested revision because Resolution No. 466/2012 does not specify the type of transportation that should be reimbursed, particularly for participants residing in municipalities within the Curitiba Metropolitan Region. **Response:** It should be clarified that, during the assessment phase referred to in ICF 1, participants will be recruited preferentially from the municipality of Curitiba. In practice, there is no compelling need to recruit participants residing in other municipalities. Additionally, participants residing in the municipality of São José dos Pinhais do not generally have differentiated transportation requirements due to the integration of the regional public transportation system. Nevertheless, the project has sufficient resources available to cover transportation expenses whenever requested by participants. This information was revised and incorporated into ICF 1. See file *TCLE_1_10_12.docx*, page 4, item m), highlighted in green. **5. Concerning Informed Consent Form 2 (ICF 2) Committee Comment:** If participants are required to travel to a Primary Health Care Unit (UBS), the possibility of reimbursement should be included in the consent form. **Response:** The Committee's concern is understood. However, we reiterate that the assessments conducted during the diagnostic phase are performed during routine visits that SUS users already make to Primary Health Care Units. Furthermore, the evaluations consist of adaptations of tests and questionnaires that are already routinely administered in these settings. Therefore, participants will not be required to attend the UBS exclusively for research purposes; rather, the assessments will occur as part of the regular

procedures that would already require such visits. Accordingly, no modifications were made to ICF 2. **6. Inclusion of Information Regarding Smartphone Use and Image Capture in Consent Forms** **Committee Comment:** The Committee requested that all informed consent forms corresponding to phases involving smartphone use and image capture explicitly include this information. **Response:** A video camera positioned perpendicular to the sagittal plane will be used only during the first phase of the study for the purpose of validating the smartphone-based assessments that will subsequently be used as movement assessment tools. It should be emphasized that the Image Use Authorization Form was included in the submitted documentation, as described in the response to Item 3. **7. Terminology Used Throughout the Project** **Committee Comment:** The Committee requested that the term *"subject"* be replaced by *"participant"* throughout the project documentation, in accordance with Brazilian National Health Council Resolution No. 466/2012. **Response:** All occurrences of the term *"subject"* were replaced with *"participant"* throughout the detailed project document, as requested. See file *Projeto_detalhado10_12.docx*, pages 35, 36, 37, 38, and 56, where the revisions are highlighted in green. **8. Concerning Research Risks** **8.1 Validation Phase Committee Comment:** During the validation phase of the study, the investigators stated that, in the event of an accident, assistance would be provided through institutional resources, including activation of emergency medical services and transportation services contracted by UFPR, followed by referral to hospitals providing public emergency care. The Committee requested clarification regarding the feasibility of such institutional support and whether formal institutional arrangements were in place. **Response:** During the validation phase, the procedures are identical to those employed in other studies previously approved by this same Research Ethics Committee and involve comparable levels of risk. It should be emphasized that the risks are minimal and are mitigated through a series of procedures designed to enhance participant safety during testing. Nevertheless, in the event of an accident, the investigators are capable of providing first aid. Among the research team members, Professor Gleber Pereira is responsible for teaching first-aid courses and possesses extensive expertise in emergency procedures. In addition, all members of the research team have received training in emergency response procedures. It should also be noted that the Department has an automated external defibrillator (AED) available for use in situations requiring resuscitation due to cardiac events. Finally, the investigators will activate emergency medical and rescue services whenever necessary, followed by referral to appropriate emergency care hospitals. We acknowledge that the institutional agreements previously established for such emergency services have unfortunately been discontinued, and the availability of alternative assistance arrangements remains uncertain. Therefore, references to services previously contracted by UFPR were removed from the detailed project description, and the information presented above was incorporated instead. See file *Projeto_detalhado10_12.docx*, page 48, highlighted in green. **8.2 Diagnostic Phase Committee Comment:** During the diagnostic phase, in which participants will be assessed at Primary Health Care Units (UBSs), the investigators stated that any adverse event would be managed by the health professionals present at the respective unit. If transfer to a medical facility becomes necessary, transportation and referral would be provided through the public emergency medical service (SAMU) and hospitals within the public health care network. Considering the declaration that the project would not generate costs and the principle that research activities should not impose additional burdens on the Brazilian Public Health System (SUS), the Committee requested revision and clarification of this item. **Response:** We understand the Committee's concern. However, the same rationale presented in Item 5 applies here, together with the

clarification provided in Item 8.1. It should be emphasized that the study aims to provide diagnostic data for SUS based on tests and examinations that are already routinely administered, albeit in a non-standardized manner. The assessments do not replace existing clinical tests and constitute routine activities within Primary Health Care Units (UBSs). It is reiterated that participants will not attend the UBSs exclusively for the purposes of this study, but rather as part of their regular health care routines that would occur regardless of the existence of the present project. The study merely provides an easy-to-use technological tool for UBSs through a different technological platform. Therefore, the project only enhances ongoing assessments and does not eliminate or modify them in a manner that would alter the risks already associated with their administration. Consequently, the minimal risks of accidents potentially associated with the execution of these tests are already present in routine UBS practice. The technology introduced by the project does not influence these risks. Therefore, the project does not impose any additional burden on the Brazilian Public Health System (SUS). Finally, it should be noted that, although the risk of adverse events is minimal, health professionals working in UBSs are adequately trained to provide first aid and arrange subsequent referral to emergency hospital services when necessary. Accordingly, no modifications were made to the study documents. **8.3 Intervention Phase** **Committee Comment:** With respect to adverse events occurring during the intervention phase, particular attention is required because the activities will be performed in participants' homes. The protocol indicates that public health services should be contacted by the participant, a family member, caregiver, or another responsible individual, as remote monitoring of activities will not be possible. The Committee requested clarification regarding how the researchers will monitor participants under these circumstances. **Response:** The intervention phase is intended as a health-promotion benefit for participants who choose to perform the exercises using the videos and instructions provided through the application. It should be emphasized that there will be no direct supervision of participants by the researchers during this phase. This is because the use of physical activity tracking systems and smartphone applications for remote exercise prescription enables remote supervision of exercise participation through a bidirectional flow of information between instructors and participants. Accordingly, every three to four months, participants who are performing the proposed physical activities and exercise sessions will be reassessed at Primary Health Care Units (UBSs) as part of their routine health evaluations in order to determine the effectiveness of the program being implemented. This information is important for the system as a whole, including participants, researchers, and Municipal Health Departments. Researcher follow-up will be conducted through the longitudinal monitoring of assessment results that will be stored over time. This information is already included in ICF-3, which recommends that participants who receive support from caregivers perform the activities under their supervision in order to facilitate participation and reduce the risk of imbalance and falls. See: *TCLE_3_10_12.docx* – information highlighted in green on page 2. *Projeto_detalhado10_12.docx* – information highlighted in green on page 50. **9. Number of Participants and Distribution Across Study Phases** **Committee Comment:** Given the large number of participants included in the study sample, the Committee requested the inclusion of a summary table detailing the distribution of participants across the various study phases and subphases. **Response:** The information was revised and updated in the proposal submission system as follows:

Group	Number of Participants	Intervention/Activity
Measurement Protocol Validation	200	Assessments during clinical tests using smartphones and gold-standard equipment
Intervention	800	Participation in exercise programs delivered through smartphones
Diagnostic Assessment	17,000	Assessments conducted through questionnaires and smartphone-based measurements

Accordingly, the Committee's request has been addressed, and the participant numbers have been revised as indicated in Item 2. **10. Inclusion Criteria Committee Comment:** For Phase 3, will participants be required to own a smartphone? At a minimum, must they possess sufficient skills to operate the application? Should these requirements be considered inclusion criteria? **Response:** Indeed, participants will need access to a smartphone or mobile device. However, the device may belong to a family member or friend who can assist them in operating the application. Therefore, access to a smartphone does not constitute a direct inclusion or exclusion criterion. Nevertheless, the consent forms and project documentation were revised to explicitly state the need for access to a smartphone. It is not feasible to establish a minimum level of smartphone proficiency as an inclusion criterion because, at advanced ages, fine motor coordination and other age-related factors may limit the use of such devices and may themselves constitute barriers to technology use. See: *TCLE_3_10_12.docx* – information highlighted in green on page 1, item b). *Projeto_detalhado10_12.docx* – information added and highlighted in green on page 41. **11. Exclusion Criteria Committee Comment:** The protocol states: *"Participants who have not signed the Free and Informed Consent Form specific to this purpose (ICF 1) will not be included." "Participants who have not signed the Free and Informed Consent Forms specific to these phases (ICF 2 and ICF 3) will not be included."* The Committee requested revision of this wording because signing the informed consent form is a mandatory prerequisite for participation in the study. Declining to sign the consent form is a participant's right and reflects a decision not to participate in the research. **Response:** The wording was revised throughout the Inclusion and Exclusion Criteria section of the detailed project and within the corresponding fields of Plataforma Brasil, as requested: *"All participants will be informed about the experimental procedures and will be required to sign the Free and Informed Consent Form specific to this phase of the study (ICF 1)." "All participants will be informed about the experimental procedures and will be required to sign the Free and Informed Consent Forms corresponding to these phases of the study (ICF 2 and ICF 3)."* See file *Projeto_detalhado10_12.docx* – information highlighted in green on page 56, Item 18. **12. Participant Transportation Committee Comment:** Transportation is anticipated during Phase 1 of the study. For the subsequent phases, will participants be assessed during their routine appointments at Primary Health Care Units (UBSs), or will appointments be scheduled specifically for research purposes? Section 19 of the protocol states: *"The remaining phases*

(diagnostic assessments) do not involve costs for participants or for institutions involved in the project (UFPR or Municipal Governments), as these evaluations constitute ongoing monitoring activities related to health status and outcomes associated with frailty, fatigue, and mobility in older adults." The Committee reiterates that, if transportation is required specifically for research purposes, reimbursement to participants must be provided. Clarification was requested. **Response:** We believe that the response provided to Item 5 adequately addresses this concern.

13. Use of Cloud-Based Data Storage **Committee Comment:** The project includes the development of a computational platform consisting of a mobile application, a cloud-based information system (web platform) with frontend and backend components, a database management system, and a set of web services made available through an Application Programming Interface (API). Accordingly, the proposed architecture includes users accessing mobile applications and the web frontend, which communicate with backend services hosted on cloud servers that provide access to database services and related functionalities. The Committee requested clarification regarding the application's privacy policy and the measures that will be adopted to protect participant data. **Response:** As described in the detailed project proposal, access to the database containing participant information will be restricted to authorized public health professionals through controlled authentication procedures based on individual login credentials and passwords. These measures are intended to ensure data security, confidentiality, and reliability.Managers of Municipal Health Departments will have access only to aggregated datasets for the purpose of identifying trends and generating projections related to the study outcomes. Because the data will be aggregated, individual participants cannot be identified. In addition to segmented reports made available to authorized users, the general public will have access to study results through a public web portal containing aggregated indicators presented in a simplified and transparent manner. Under no circumstances will this public information allow the identification of individual participants. It should be emphasized that one of the primary objectives of the project is precisely to provide health-related information both to participants (through individualized access linked to their personal identification number) and to public health professionals (through controlled access levels), enabling the implementation of preventive or corrective actions aimed at improving the health status of the population and addressing identified risks and/or health problems. The public health professionals referred to in the project are those designated by the Primary Health Care Units (UBSs) who will receive specific training. These professionals already have authorized access to the medical records of SUS users. The application will provide UBS personnel with relevant information regarding the users served by their respective health units, thereby enabling closer monitoring of individuals receiving care through SUS. Finally, from a technical standpoint, the data will be protected through cybersecurity measures designed to prevent unauthorized access, hacking attempts, and other forms of cyberattacks. Municipal employees already have access to SUS patient medical records, and it is assumed that access to the data generated by this study will not differ from the access already granted through existing health information systems. It is reiterated that the tests included in the present study are already routinely performed in clinical practice; however, they are currently conducted without systematic data management or advanced analysis of their outcomes. Considering the above, no modifications were made to the study documentation.

14. Ownership and Confidentiality of Information **Committee Comment:** The protocol states: *"In the subsequent phases of the study (beginning with Phase 2), health professionals from Primary Health Care Units will also have access to test results and may contact participants to provide guidance when necessary. Assessment results collected at Primary*

Health Care Units will be stored in a cloud-based repository to enable longitudinal follow-up. Furthermore, the evaluation of large populations will allow the creation of a database capable of providing reference values that may help public authorities better understand and address different realities, thereby promoting equity and comprehensiveness in care for the older adult population, which is one of the objectives of this study." The Committee noted that access to research data should be restricted to members of the research team. Therefore, clarification was requested regarding who these health professionals are, whether they would be formally included in Plataforma Brasil through a project amendment, and whether they would assume the responsibilities described in a specific agreement. The Committee also requested clarification regarding data anonymization procedures and the mechanisms that will be adopted to maintain confidentiality. **Response:** Given the large number of health professionals working in Primary Health Care Units across the various municipalities that may participate in this project, it is not feasible to include all of them in Plataforma Brasil through a project amendment. Therefore, only trained public health professionals will be authorized to access the database through secure login credentials and passwords (possibly the same credentials already used to access other municipal health information systems) in order to perform their professional duties related to monitoring and follow-up of participants' health conditions to ensure the reliability and appropriate use of the information. In fact, these public health professionals must have access to each participant's individual parameters in order to provide appropriate guidance and implement any necessary interventions. It should also be emphasized that health professionals working in Primary Health Care Units (UBSs) already have unrestricted access to the medical records of SUS users. Therefore, no explicit need was identified to create additional instruments that would merely duplicate existing controls over sensitive health information. Once again, it is reiterated that the tests included in this study are already routinely performed by UBSs. The data generated are of the same nature as those already collected in clinical practice, differing only in the method by which they are obtained and stored. Consequently, public health professionals already possess access rights to this type of information. Considering the above, no modifications were made to the study documentation. **15. Vulnerable Groups Committee Comment:** The project indicated that the inclusion of vulnerable groups was "not applicable." The Committee requested justification for the inclusion of older adults aged 60 to 80 years, who may present reduced autonomy or experience changes in their clinical condition during the course of the study. **Response:** The Committee's observation is acknowledged. The wording in Item 15 of the detailed project was revised to address this concern as follows: *"The project involves older adults who may be considered vulnerable due to mobility impairments, loss of autonomy, and/or situations of physical and/or mental incapacity that may already be present at the beginning of the study or may develop over time, resulting in increased vulnerability."* See file *Projeto_detalhado10_12.docx* – information added and highlighted in green on page 55, Item 15. **16. Budget Committee Comment:** The budget includes expenditures for fruit and water for participants. The Committee requested clarification regarding when these items will be provided. **Response:** Fruit will be offered at the beginning and end of data collection sessions, while water will be available *ad libitum*. It should be emphasized that these items will be provided only during Phase 1 of the study, which will be conducted at the facilities of the Department of Physical Education of the Federal University of Paraná (UFPR). This practice is a courtesy routinely used in studies conducted by the Center for Motor Behavior Studies (CECOM) to improve participant comfort and well-being during assessment procedures. **17. Budget Committee Comment:** The Committee requested the inclusion of a detailed budget consistent

with the resources awarded by FINEP, specifying the corresponding expenditures rather than presenting only the brief summary contained in the project proposal. **Response:** The project has already undergone merit review by FINEP, UFPR, and FUNPAR and was considered adequate. Budgetary merit analysis falls within the scope of proposal evaluation and judgment conducted by these institutions. We emphasize that the project budget includes: Scholarships and student support; Insurance expenses for students involved in the project; Acquisition of permanent equipment; Contracted services for software and application development; Operating and consumable expenses; Travel expenses for dissemination of study results; National and international travel to conferences and scientific meetings; Administrative and management reimbursement costs payable to FUNPAR. For purposes of transparency and accuracy, screenshots of the project budget were provided. It should be noted that a substantial proportion of the project's resources is allocated to student scholarships. In light of the above, the pending issues identified by the Committee were considered satisfactorily addressed.

Final Considerations of the Research Ethics Committee (CEP)

01. The Committee requests the submission of **semiannual progress reports** (every six months following approval of the study) and a **final report** describing the progress of the research, as well as any information regarding protocol modifications, cancellation, completion of the study, and the destination of the knowledge generated. These reports must be submitted through **Plataforma Brasil** using the **NOTIFICATION** submission mode. For the next report, investigators should use the updated partial report template (April 2022 version).

02. Any additional modifications to the protocol, as well as requests for extensions of the study period, must be submitted through the **AMENDMENT** submission mode (*Emenda*). Investigators are reminded that the research schedule registered in Plataforma Brasil must be updated before submitting a request to extend the study period. A template amendment letter is available on the Committee's website: www.cometica.ufpr.br. Submission of this document is mandatory.

03. Important (when applicable): Any pending issues related to participating institutions (*co-participant institutions*) must be addressed by the responsible institution through Plataforma Brasil in accordance with the applicable regulatory procedures through the account of the Principal Investigator. For projects involving co-participating institutions that are also required to submit semiannual reports, such reports must be submitted as **Notifications** through the login credentials of the Principal Investigator, under the CAAE corresponding to the respective co-participating institution, after submission of the report to the proposing institution.

04. The **CAAE number** and the **number of the approved Ethics Committee Opinion** must be included in all **Informed Consent Forms (ICF/TCLE)** and **Assent Forms (TALE)** before their use with participants.

Documents Submitted and Reviewed by the Research Ethics Committee

Document Type	File Name	Submission Date	Author	Status
Basic Project Information	PB_INFORMAÇÕES_BÁSICAS_DO_PROJETO_2431843.pdf	11-Dec-2024, 08:19:10	—	Accepted
Other Document	RESPOSTA_ETICA_10_12.docx	10-Dec-2024, 16:56:43	Roberta Castilhos Detanico Bohrer	Accepted
Detailed Project / Investigator's Brochure	Projeto_detalhado10_12.docx	10-Dec-2024, 16:56:01	Roberta Castilhos Detanico Bohrer	Accepted
Informed Consent Form / Assent Form / Justification of Absence	TCLE_3_10_12.docx	10-Dec-2024, 16:55:48	Roberta Castilhos Detanico Bohrer	Accepted
Informed Consent Form / Assent Form / Justification of Absence	TCLE_1_10_12.docx	10-Dec-2024, 16:55:35	Roberta Castilhos Detanico Bohrer	Accepted
Cover Sheet	Folha_rosto_ass_05_12.pdf	10-Dec-2024, 16:55:01	Roberta Castilhos Detanico Bohrer	Accepted
Other Document	Termo_de_imagem.docx	09-Dec-2024, 10:19:11	Roberta Castilhos Detanico Bohrer	Accepted
Informed Consent Form / Assent Form / Justification of Absence	TCLE_2_07_10.docx	14-Oct-2024, 13:58:47	Roberta Castilhos Detanico Bohrer	Accepted

Other Document	Requerimento_apreciacao_projetos.pdf	10-Oct-2024, 11:54:08	Roberta Castilhos Detanico Bohrer	Accepted
Other Document	Declaracao_ausencia_custos_SMS.pdf	10-Oct-2024, 11:53:32	Roberta Castilhos Detanico Bohrer	Accepted
Other Document	Carta_Encaminhamento_0710.pdf	10-Oct-2024, 11:52:00	Roberta Castilhos Detanico Bohrer	Accepted
Other Document	Analise_Merito_ass.pdf	10-Oct-2024, 11:50:31	Roberta Castilhos Detanico Bohrer	Accepted
Statement of Agreement	Concordancia_servicos_envolvidos.pdf	10-Oct-2024, 11:50:06	Roberta Castilhos Detanico Bohrer	Accepted
Research Timeline	Cronograma.pdf	10-Oct-2024, 11:48:51	Roberta Castilhos Detanico Bohrer	Accepted
Other Document	Checklist.docx	04-Oct-2024, 11:00:19	Roberta Castilhos Detanico Bohrer	Accepted
Sponsor Declaration	APROVACAO_FINEP.pdf	04-Oct-2024, 10:49:36	Roberta Castilhos Detanico Bohrer	Accepted
Budget	Orcamento.docx	04-Oct-2024, 10:46:06	Roberta Castilhos Detanico Bohrer	Accepted
Other Document	APROVACOES.pdf	04-Oct-2024, 10:38:12	Roberta Castilhos Detanico Bohrer	Accepted
Other Document	Compromisso_EquipePesquisa.pdf	04-Oct-2024, 10:34:46	Roberta Castilhos Detanico Bohrer	Accepted
Other Document	Carta_SMS_assinada.pdf	03-Oct-2024, 17:31:20	Roberta Castilhos Detanico Bohrer	
Other Document	Carta_SMS_assinada.pdf	03-Oct-2024, 17:31:20	Roberta Castilhos Detanico Bohrer	Accepted

Ethics Committee Decision

Opinion Status: Approved

Requires CONEP Review: No

The Research Ethics Committee concluded that the project meets the applicable ethical and regulatory requirements and approved its implementation as submitted and subsequently revised in response to the Committee's requests.

The Committee determined that all pending issues had been adequately addressed by the research team and that the proposed procedures present acceptable and appropriately managed risks in light of the expected scientific and social benefits.

Curitiba, Paraná, Brazil
December 19, 2024

Signed by:

Ida Cristina Gubert
Chairperson (Coordinator)
Research Ethics Committee (CEP)
Federal University of Paraná (UFPR)

PARECER CONSUBSTANCIADO DO CEP

DADOS DO PROJETO DE PESQUISA

Título da Pesquisa: Tecnologia Assistiva na Avaliação Clínico-funcional Integrada e Prescrição de Exercícios para Pessoas Idosas Usuárias do SUS

Pesquisador: André Luiz Felix Rodacki

Área Temática:

Versão: 2

CAAE: 83996424.4.0000.0102

Instituição Proponente: Departamento de Educação Física - UFPR

Patrocinador Principal: Financiadora de Estudos e Projetos - FINEP

DADOS DO PARECER

Número do Parecer: 7.310.210

Apresentação do Projeto:

Este parecer foi elaborado com base nas informações contidas na Plataforma Brasil.

Projeto intitulado Tecnologia Assistiva na Avaliação Clínico-funcional integrada e prescrição de exercícios para pessoas idosas usuárias do SUS, de autoria do Professor André Luiz Felix Rodacki, proveniente do Departamento de Educação Física da UFPR, com colaboração dos Professores Dr. Alessandro Brawerman, Dr. Dieval Guiselini, Dr. Gleber Pereira, Dr. José Luiz Padilha da Silva, Dra. Luciana Puchalski Kalinke, Dra. Natália Boneti Moreira, Dr. Paulo Cesar Barauce Bento e Dra. Roberta Castilhos Detanico Bohrer. Trata-se de projeto aprovado pela FINEP, chamada Pública ¿MCTI/FINEP/AÇÃO TRANSVERSAL - TECNOLOGIA ASSISTIVA, (NÚMERO DE REFERÊNCIA 2137/22) na seleção pública de propostas para o apoio financeiro a projetos institucionais para a estruturação do sistema nacional de laboratórios de tecnologia assistiva (sisassistiva-MCTI). A aprovação da proposta foi vinculada a um aporte de recursos de R$3.903.657,79 (três milhões, Novecentos e três mil, seiscentos e cinquenta e sete reais e setenta e nove centavos). O orçamento da pesquisa é de R$8.400,00.

O período de realização da pesquisa é de novembro de 2024 a dezembro de 2027. A população de estudo descrita na folha de rosto é de 10 000 participantes idosos entre 60 e 80 anos. O estudo está composto por fases, que compreendem: a) validação de testes (transposição de testes clínicos e questionários) para ambiente eletrônico em smartphone b) diagnóstico de

idosos usuários das Unidades Básicas de Saúde a partir de um aplicativo especialmente desenvolvido para essa finalidade e c) oferta de um programa de intervenção especificamente desenhado para idosos, através do smartphone. O local de pesquisa na Fase 1 do estudo será o Centro de Estudo do Comportamento Motor (CECOM), localizado no Departamento de Educação Física, no Setor de Ciências Biológicas, no Centro Politécnico da Universidade Federal do Paraná (UFPR). As fases subsequentes, especificamente as Fases 2 e 4 do estudo (Estudo Piloto e Treinamento), serão realizadas nas Unidades de Saúde indicadas pelas Prefeituras participantes deste projeto.

De acordo com o exposto pelos pesquisadores, a primeira fase denominada de validação buscará identificar se as medidas obtidas por sensores de smartphones (acelerômetros e giroscópios) podem ser comparadas àquelas obtidas em equipamentos considerados gold-standard. Estima-se que para esta fase sejam suficientes aproximadamente 200 participantes. Uma vez que as medidas representativas e válidas referentes a um conjunto de testes possa ser coletado em smartphones, os algoritmos serão congregados em um aplicativo que será capaz de auxiliar os profissionais das unidades básicas de saúde a aplicar questionários e realizar testes físicos. Uma vez que o aplicativo seja disponibilizado, será feito um estudo piloto para identificar sua capacidade de realizar medidas em idosos em um conjunto reduzido de Unidades Básicas de Saúde. Para que as medidas sejam confiáveis, os profissionais que atuam nas Unidades Básicas de Saúde serão qualificados para operar o aplicativo. Estima-se que para essa fase sejam analisados aproximadamente 8000 idosos. Uma vez resolvidas as questões e dificuldades do aplicativo durante a fase piloto, ocorrerá uma coleta de dados em larga escala em todas as Unidades Básicas de Saúde, de forma com que os dados possam ser utilizados para que processos de Inteligência Artificial possa realizar análises que identifiquem e possam identificar se os questionários e testes físicos podem auxiliar no diagnóstico dos principais desfechos do estudo. Caso isso seja possível, o banco de dados poderá ser utilizado para mapeamentos dos indivíduos com maior predisposição (risco aumentado) aos desfechos do estudo, o que permitirá que a Secretaria Municipal de Saúde possa realizar ações preventivas adequadas. Os métodos propostos para as avaliações de idosos são amplamente utilizados, conforme descrito na literatura. Finalmente, a última fase do projeto envolve a oferta de atividades físicas especificamente desenvolvidas para idosos, as quais serão realizadas a partir dos smartphones. O programa de atividades físicas é relevante pois permitirá com que idosos sedentários possam incrementar seus níveis de atividade física e melhorar seus indicadores de

saúde de forma atrativa, gratuita e com baixa demanda de recursos físicos (cadeiras, toalhas, garrafas PET, etc.). Além disso, os idosos podem ser beneficiar de tais programas uma vez que não existe demanda de deslocamento para a realização das atividades que podem ser feitas em regime domiciliar. O programa de treinamento seguirá diretrizes previamente publicadas e bem definidas.

Dentre os resultados, espera-se fornecer ainda um programa de atividades físicas que possa ser executado com baixo custo e que seja efetivo para incrementar o nível de atividade física entre os idosos e incrementar seus estados de saúde, conforme mensurações diagnósticas propostas. Os dados coletados serão submetidos a análises que envolvem inteligência artificial para a predição dos estados de saúde (fragilidade, fadiga, mobilidade e quedas). Desta forma, ficam evidenciados os benefícios para a Ciência e para a Sociedade. Por outro lado, os participantes poderão ter seus testes disponibilizados de forma rápida e acessível em seus smartphones de forma que os valores de seu desempenho podem ser compreendidos por valores referenciais obtidos do estudo. Além disso, os dados serão armazenados de forma com que os participantes (e os responsáveis pelo acompanhamento clínico) poderão acompanhar os estados de saúde ao longo do tempo. Finalmente, os participantes terão a oportunidade de se beneficiar das atividades físicas propostas pelo aplicativo, as quais serão ofertadas de forma gratuita.

Critério de Inclusão: Para efeitos de validação dos testes funcionais (Fase 1 do estudo), serão aplicados alguns critérios de inclusão e exclusão. Serão incluídos na pesquisa indivíduos com idade superior a 60 anos (entre 60 e 80 anos), de ambos os sexos, que se voluntariem a participar do estudo. Para as fases subsequentes do estudo, serão incluídos indivíduos com idade igual ou superior a 60 anos (entre 60 e 80 anos), de ambos os sexos, que se voluntariem a participar do estudo. Não existem restrições para a participação de nenhum teste associado ao projeto visto que todos são de fácil execução e constituem prática clínica corrente em geriatria. Em geral, também não existem restrições a diferentes estados de saúde, visto que os testes propostos são aplicados para tais diagnósticos. Critério de Exclusão: Serão excluídos da pesquisa indivíduos com alterações cognitivas e neurológicas severas que impossibilitem a realização dos testes funcionais, preenchimento dos questionários ou compreensão das orientações. Todos os critérios serão identificados por meio da triagem inicial realizada pelo pesquisador. Não serão incluídos participantes que não tenham assinado o Termo de Consentimento Livre e Esclarecido, específico para essa finalidade (TCLE 1). Não serão incluídos participantes que não tenham

assinado o Termo de Consentimento Livre e Esclarecido, específico para essa finalidade (TCLE 2 e TCLE 3).

Objetivo da Pesquisa:

Objetivo Primário:

A proposta visa estabelecer uma avaliação clínico-funcional (funcionalidade, fatores perceptivos-cognitivos e risco de quedas) padronizada e integrada nos diferentes serviços de saúde a partir de medidas obtidas através de sensores de smartphones, bem como determinar a efetividade de um programa de exercícios domiciliares aplicados por tecnologia assistiva para pessoas idosas usuárias do SUS.

Objetivo Secundário:

1. Implementar no sistema/aplicativo questionários para avaliar o histórico de quedas, o declínio cognitivo, o estado de fragilidade, o medo de cair, o estado de fadiga e o nível de atividade física e comportamento sedentário;
2. Desenvolver e validar algoritmos de testes de desempenho físicofuncional a partir de smartphones a fim de identificar redução de mobilidade e risco de quedas;
3. Identificar possíveis relações diagnósticas e de acompanhamento do estado de saúde e do desenvolvimento de doenças a partir dos dados sociodemográficos e das avaliações clínicas e físicofuncionais;
4. Compreender a transição dos estados de saúde na terceira idade a partir do histórico das avaliações dos participantes;
5. Identificar valores referenciais para os testes clínicos fisico-funcionais considerando diferentes faixas etárias e características associadas à saúde;
6. Identificar os níveis de fadiga de idosos sobreviventes de câncer;
7. Propor um programa de atividade física monitorado à distância por intermédio de smartphones;
8. Avaliar a qualidade de vida de idosos sobreviventes de câncer durante a participação do programa de atividade física monitorado a distância;
9. Analisar a efetividade de um programa de exercícios físicos domiciliares monitorado à distância por intermédio de smartphones sobre os aspectos da mobilidade e perceptivo-cognitivos de idosos.

Avaliação dos Riscos e Benefícios:

Riscos: Ao responder os questionários é possível que os participantes sintam algum

constrangimento, em razão de perguntas de cunho pessoal, como idade, nível de escolaridade e número de doenças, por exemplo. Todavia, tais questionamentos são básicos em todos os tipos de investigação ou ainda quando do registro dos participantes em programas de intervenção. Ao realizar os testes físicos é possível que os participantes sintam cansaço muito leve ou em alguns casos pode sentir uma leve dor muscular tardia. O cansaço e a dor muscular tardia são respostas normais e devem desaparecer rapidamente. Para os participantes do programa de exercícios físicos domiciliares, é possível que apresentem cansaço e dores musculares e articulares durante e/ou após os exercícios devido ao esforço inerente à prática de exercícios, especialmente para aquelas pessoas que não estão acostumadas. É possível ainda que experimentem algum desequilíbrio ou até mesmo alguma queda, caso não sigam as recomendações de prevenção/segurança. Instruções iniciais a cada sessão sobre segurança serão providas nos aplicativos enfatizando o uso de materiais seguros e que provejam estabilidade (ex. cadeiras) durante as atividades. Ainda que tais riscos sejam inerentes a toda e qualquer atividade de avaliação e programas de exercício, são raros os casos em que os participantes possam sofrem intercorrências. Há que se destacar o fato que os agentes dos postos de saúde serão treinados para a aplicação de testes nas UBSs. Ademais, todos os testes são clinicamente seguros e têm sido aplicados em larga escala em idosos no país e internacionalmente.

Benefícios: A partir dos resultados encontrados no estudo, como benefício direto e individual, os participantes terão resultados acerca da sua capacidade físicofuncional, disponibilizados no aplicativo, considerando a faixa etária. Esses benefícios podem proporcionar um melhor entendimento sobre suas habilidades no desempenho de atividades funcionais. Isso poderá motivá-lo a adotar um estilo de vida mais ativo e engajar-se regularmente em exercícios físicos. Para os idosos participantes do programa de exercícios físicos domiciliares espera-se como benefício direto e individual, ganhos na funcionalidade, o que consequentemente melhora a capacidade de realizar atividades da vida diária e diminui o risco de quedas.
De forma indireta, este estudo poderá gerar um impacto positivo no campo científico uma vez que avaliações clínico-funcionais por meio de tecnologias assistivas (smartphones) possam substituir equipamentos de alto custo e difícil acessibilidade para a comunidade. Isso acarretará benefícios para os gestores em saúde, pois permitirá abrangência das avaliações bem como a possibilidade de acompanhamento dos estados de saúde da comunidade de

usuários do SUS. Adicionalmente, a proposição de programas de exercícios físicos domiciliares monitorado à distância por intermédio de smartphones permite maior abrangência e acessibilidade para que indivíduos possam exercitar-se em casa, em qualquer horário e sem a necessidade ou dependência de terceiros para locomover-se até locais de prática específicos. Tais aspectos constituem estratégias de promoção e prevenção da saúde da crescente população idosa. Espera-se que através desse estudo, seja possível fornecer ferramentas diagnósticas para os serviços públicos de saúde. Os avanços nas ferramentas se apresentam na transposição de vários questionários para o formato eletrônico e na análise de valores referenciais locais, visto que não são conhecidos. Além disso, o armazenamento dos dados possibilitará efetuar acompanhamentos longitudinais dos avaliados e observar as transições de seus estados (fragilidade, fadiga, mobilidade, etc.). Por outro lado, espera-se incrementar as ferramentas tecnológicas disponíveis, as quais podem permitir universalizar vários testes clínicos de forma com que se possa estabelecer valores normativos para a comunidade. Da mesma forma, espera-se poder fornecer aos serviços públicos formas acuradas de medir o desempenho em vários testes físicos de forma objetiva e com custos reduzidos, uma vez que os equipamentos considerados como padrão ouro (ex. plataformas de força) são pouco acessíveis para aqueles que atuam em unidades de saúde. Dentre os resultados, espera-se fornecer ainda um programa de atividades físicas que possa ser executado com baixo custo e que seja efetivo para incrementar o nível de atividade física entre os idosos e incrementar seus estados de saúde, conforme mensurações diagnósticas propostas. Os dados do estudo podem fornecer subsídios aos gestores públicos para que política de saúde sejam incrementadas ou ainda revisadas de forma que suas decisões sejam fundamentadas em parâmetros objetivamente mensurados. Os resultados do estudo podem servir como referência para que outros municípios adotem estratégias de monitoramento e intervenção comparáveis. Espera-se ainda que o estudo possa auxiliar na publicação de materiais bibliográficos (artigos, capítulos, livros, materiais instrucionais) e possam contribuir para a formação de profissionais mais bem qualificados na atuação dos processos de saúde relacionados aos idosos que recorrem aos serviços de saúde das unidades básicas de saúde.

Comentários e Considerações sobre a Pesquisa:

Pesquisa bem delineada e de alto impacto social. Além dos benefícios diretos aos participantes, destacam-se aqueles indiretos. Trata-se de pesquisa com intervenção, que visa a obtenção de ferramentas diagnósticas para os serviços públicos de saúde e de programa de atividades

físicas que possa ser executado com baixo custo e que seja efetivo para incrementar o nível de atividade física entre os idosos e incrementar seus estados de saúde, conforme mensurações diagnósticas propostas.

Considerações sobre os Termos de apresentação obrigatória:

Os termos foram apresentandos no atendimento às pendências.

Recomendações:

Não há.

Conclusões ou Pendências e Lista de Inadequações:

Considerando que as justificativas apresentadas pelos pesquisadores complementam a análise ética do estudo, e confirmam o compromisso ético dos pesquisadores na condução do estudo, transcrevem-se as justificativas apresentadas.

1- Termos de apresentação obrigatória:- O estudo prevê como local as Secretarias Municipais de Saúde de Curitiba e da Região Metropolitana. Contudo, constam as cartas de anuência somente da SMS Curitiba, e demais secretarias não foram indicadas como coparticipantes. Solicita-se esclarecer.

Resposta: O parecer está correto em sua análise. Todavia, o momento de troca de gestão nas prefeituras municipais e dos gestores das secretarias municipais de saúde não permite que as prefeituras envolvidas efetivamente emitam parecer de adesão à proposta, a qual depende de aprovação local. No momento, aguardamos que as novas gestões (2025) analisem a proposta e confirmem a intenção indicada ao longo das tratativas de 2024. Desta forma, faremos aditivos das cartas de anuência das Prefeituras que aderirem à proposta ao longo do ano de 2025.

2. - Em relação à folha de rosto: foram indicados 10.000 participantes, contudo no item 6.1.4 Treinamento dos profissionais das UBSs consta que estima-se que nessa fase (1º ano de disponibilização), pelo menos 10% dos idosos dos municípios de Curitiba e Região Metropolitana sejam avaliados (aproximadamente 18.000 idosos), sendo tais números ampliados ao longo dos demais anos. Solicita-se esclarecer a amostra e se necessário, alterar a folha de rosto.

Resposta: O parecerista está correto. O número de participantes foi ajustado conforme a

estimativa de cerca de 18.000 idosos avaliados no município de Curitiba e Região Metropolitana. Segue nova folha de rosto ajustada. Arquivo: ¿Folha_rosto_ass_05_12.pdf¿.

3 - Em relação ao uso de imagem: consta no projeto - Para o teste, os participantes segurarão o smartphone na altura do osso esterno (centro do peito) com ambas as mãos e a partir da posição sentada, iniciarão o teste. Simultaneamente, haverá uma câmera de vídeo posicionada perpendicularmente ao plano sagital de movimento com o foco direcionado à altura do ombro. Solicita-se a inclusão do Termo de uso de imagem e/ou voz e /ou prontuários, conforme modelo 15 do site do CEP/SD.

Resposta: Esclarecemos que as imagens realizadas no plano sagital visam fornecer as coordenadas de pontos corporais, as quais serão usadas para calcular os parâmetros do movimento. Portanto, destaca-se que não haverá veiculação de imagens em que os sujeitos possam ser identificados em ambientes fora daqueles pertinentes à análise. Os vídeos são de circulação estritamente interna para processamento de dados, ou seja, em ambiente restrito. Ainda assim, e para acomodar as pertinentes preocupações do Comitê, foi inserido o termo de uso de imagem como um documento adicional, conforme sugerido. Arquivo: ¿Termo_de_imagem.docx¿.

4 - Em relação ao TCLE 1: m) As despesas necessárias para a realização da pesquisa (avaliações, materiais descartáveis, etc.) não são de sua responsabilidade e pela sua participação no estudo o(a) senhor(a) você não receberá qualquer valor em dinheiro. Caso necessário, será realizado um ressarcimento, via cartão transporte utilizado para o transporte público, dos valores de deslocamento
dos participantes para o Departamento de Educação Física, no campus do Centro Politécnico da UFPR. (Item II.21, e item IV.3, subitem g, Resol. 466/2012). Solicita-se rever, pois a Resolução n.466/2012 não estabelece o tipo de transporte que deverá ser ressarcido, especialmente nos casos em que o participante resida na região metropolitana de Curitiba.

Resposta: Cabe esclarecer que na fase de avaliação, a que remete o TCLE 1, os participantes serão recrutados preferencialmente da cidade de Curitiba. De fato, não há necessidade imperiosa de recrutar participantes que residam em outros Municípios. Pesa o fato de que participantes do Município de São José dos Pinhais não possuem demandas diferenciadas em

função da integração do sistema de transporte. Ainda assim, o projeto possui recursos para a cobertura de despesas conforme demandado pelos participantes. Tais informações foram ajustadas no TCLE 1.
Vide documento arquivo TCLE_1_10_12.docx ¿ p. 4 ¿ item m), destacado em verde.

5 - Em relação ao TCLE 2 caso seja necessário o deslocamento do participante à UBS, incluir a possibilidade de ressarcimento.

Resposta: Entende-se a preocupação do parecer. Entretanto, reiteramos que as análises da fase diagnóstica decorrem das visitas regularmente feitas pelos usuários do SUS às UBS. Reitera-se que as avaliações realizadas constituem ajustes dos testes e questionários que já são rotineiramente aplicados. Desta forma, a visita dos participantes à UBS não será realizada exclusivamente para fins de avaliação, mas como parte dos procedimentos frequentes que já demandariam tal visita. Dessa forma, nenhum ajuste foi realizado no TCLE 2.

6 - Em todos os TCLEs das fases em que houver relação com o uso de smartfone e captação de imagem, solicita-se a inclusão dessa informação.

Resposta: Apenas na primeira fase será utilizada uma câmera de vídeo posicionada perpendicularmente ao plano sagital para fins de validação do uso dos smartphones a serem utilizados posteriormente como instrumento de avaliação do movimento. Ressalta-se que o Termo de uso de imagem foi incluído na documentação enviada, conforme indicado na resposta ao item 3.

7 - Ao longo do projeto detalhado, solicita-se a substituição do termo sujeito por participante, conforme a Resolução CNS n. 466/2012.

Resposta: A substituição de todos os termos ¿sujeito¿ por ¿participante¿ foi realizada em todo o projeto detalhado, conforme solicitado.
Arquivo ¿Projeto_detalhado10_12.docx¿ - Páginas 35, 36, 37, 38 e 56, conforme destacado na cor verde.

8 - Em relação aos riscos da pesquisa:
8.1- 1) na fase de validação do estudo, os pesquisadores declararam que em caso de acidente, o socorro será fornecido pelos recursos disponíveis pela Instituição que consiste no acionamento de sistema de emergência médica e remoção contratado pela UFPR e encaminhamento a hospitais que disponham de serviços públicos de atendimento de urgências médicas. Solicita-se esclarecer se o custo da Universidade é viável e se há apoio institucional formalizado para tal.

Resposta: Na fase de validação, os procedimentos são idênticos e envolvem os mesmos riscos comparados a outros estudos já aprovados por este mesmo Comitê de Ética. Destaca-se que os riscos são mínimos e mitigados por um conjunto de procedimentos que aumentam a segurança durante os testes. Todavia, em caso de acidentes, os pesquisadores poderão prestar os primeiros socorros. Dentre a equipe de pesquisadores, o Prof. Gleber Pereira é responsável pela disciplina de primeiros socorros e possui pleno conhecimento de tais procedimentos. Ademais, todos os membros receberam formação acerca dos procedimentos para emergências. Destaca-se ainda que existe no departamento um desfibrilador que pode ser utilizado em casos que demandem reanimação por episódio cardíaco. Finalmente, cabe destacar que os pesquisadores farão o acionamento dos serviços de emergência/resgate com subsequente encaminhamento aos hospitais de pronto atendimento de emergências.

Reconhecemos que, infelizmente, os convênios para tais atendimentos tenham sido descontinuados, sendo desconhecidos a vigência de novas formas de assistência. Desta forma, removemos a informação sobre os serviços contratados pala UFPR no projeto detalhado e incluimos informações conforme descrito acima.
Vide documento do Arquivo ¿Projeto_detalhado10_12.docx¿ ¿ p. 48 em verde.

8.2 2) Já na fase de diagnóstico - em que os participantes serão avaliados nas UBSs, os pesquisadores referem que qualquer intercorrência será atendida pelos profissionais de saúde presentes naquela unidade. Frente a necessidade de remoção para serviços médicos, a remoção e o encaminhamento se darão pelos serviços públicos do SAMU e pelos hospitais da rede pública de atendimento. Considerando a carta de ausência de custos, e que as pesquisas não podem onerar o SUS, solicita-se revisão deste item.

Resposta: Entendemos a preocupação do parecer. Todavia, a mesma argumentação apresentada no item 5 é novamente aplicada, adicionada ao esclarecimento apresentado no item 8.1. Cabe destacar que o estudo visa prover dados diagnósticos para o SUS a partir dos testes e exames que já são aplicados e de forma não estruturada. As avaliações não substituem os testes clínicos que já são usados e constituem atividades corriqueiras nas UBSs. Reitera-se que os participantes não irão às UBSs exclusivamente para essa finalidade, mas como parte da rotina de exames que já ocorre sem a existência do presente projeto. O estudo apenas provê uma ferramenta de fácil uso e aplicação para as UBSs por uma tecnologia diferente. Portanto, o estudo apenas aprimora os testes em curso, mas não os exclui ou os modifica de forma que os riscos presentes sejam alterados. Logo, os riscos de acidentes (mínimos) que porventura possam estar associados à execução dos testes já estão presentes e são realizados rotineiramente pelas UBSs. Portanto, os testes não são influenciados pela tecnologia presente. Logo, não existe ônus para o SUS em decorrência do presente projeto. Finalmente, destaca-se que, ainda que os riscos de intercorrências sejam mínimos, os profissionais de saúde que atuam nas UBSs são bastante capacitados para que os primeiros socorros sejam prestados com subsequente encaminhamento para o pronto atendimento de emergência em Hospitais.
Dessa forma, não houve alterações nos textos.

8.3. 3) E, em casos de intercorrências na fase de intervenção merece destaque visto que as atividades são executadas no ambiente doméstico definem que os serviços de saúde públicos deverão ser acionados pelo participante, familiar, cuidador ou congênere, uma vez que não será possível efetuar o monitoramento remoto das atividades. Solicita-se esclarecer como ocorrerá o acompanhamento dos pesquisadores nessa condição.

Resposta: A fase de intervenção constitui-se como um benefício de promoção de saúde aos participantes que desejem realizar os exercícios a partir dos vídeos e orientações dados via aplicativo. Salienta-se que nessa fase não haverá qualquer acompanhamento direto aos participantes por parte dos pesquisadores, pois o uso de rastreadores de atividade física e de aplicativos para prescrição de exercícios à distância permitem uma supervisão remota da realização dos exercícios com o fluxo de informações sobre a realização dos exercícios bidirecional, tanto para os professores quanto para os praticantes. Dessa forma, a cada 3-4 meses os participantes que estiverem realizando as atividades e exercícios físicos propostos

poderão ser avaliados nas UBSs ¿ como parte de suas rotinas de avaliação ¿ para fins de avaliar a efetividade do programa que está sendo aplicado, o que é importante para o sistema como um todo (participantes-pesquisadores-Secretarias de Saúde). O acompanhamento dos pesquisadores será dado pelo seguimento dos resultados dos testes que serão armazenados ao longo do tempo (longitudinalmente). No TCLE-3 já consta essa informação, recomendando que para aqueles participantes que contem com apoio de cuidadores, as atividades sejam acompanhadas por estas pessoas para facilitar as atividades e reduzir o risco de desequilíbrios e quedas.
Arquivo ¿TCLE_3_10_12.docx¿ ¿ informação destacada em verde na pág. 2
Arquivo ¿Projeto_detalhado10_12.docx¿ ¿ informação destacada em verde na pág. 50

9. - Em relação ao número de participantes e distribuição nas fases e ações: Considerando o número elevado de participantes na amostra, com a finalidade de clarear a distribuição desses no decorrer do estudo, solicita-se a inclusão de um quadro síntese que detalhe cada fase e suas subfases.

Resposta: A informação reaparece ajustada na tela do sistema de submissão da proposta, conforme segue abaixo

Grupo   Número de Indivíduos   Intervenção a serem realizadas
Validação de Protocolos de Medida 200   Medidas durante testes clínicos por smartphones e padrão ouro
Intervenção   800   Participação em programas de exercícios aplicados via smartphone
Diagnóstico   17.000   Mensuração via questionários e smartphones

Desta forma, atende-se a demanda do parecer e ajusta-se o número de participantes, conforme indicado no item 2.

10- Em relação aos critérios de inclusão: para a fase 3 o participante precisará possuir um smartphone? E ao mínimo, ter habilidades de manuseio do aplicativo. Esses são critérios de inclusão?

Resposta: De fato, o participante de fato precisará ter acesso a um smartphone/dispositivo móvel. Contudo, este poderá ser de um familiar ou amigo que poderá auxiliá-lo no manuseio do aplicativo. Assim, não constitui critério direto de inclusão e/ou exclusão. Todavia, ajustamos os termos e o projeto para informar essa necessidade de acesso a um smartphone. Não há como controlar o nível de habilidade para o uso de smartphones dado que em idades muito avançadas a coordenação fina e outros aspectos limitam uso de tais ferramentas, o que pode constituir uma barreira.
Vide:
Arquivo ¿TCLE_3_10_12.docx¿ ¿ informação destacada em verde na pág. 1 item b).
Arquivo ¿Projeto_detalhado10_12.docx¿ ¿ informação adicionada e destacada em verde na pág. 41

11 - Em relação aos critérios de exclusão: Não serão incluídos participantes que não tenham assinado o Termo de Consentimento Livre e Esclarecido, específico para essa finalidade (TCLE 1). Não serão incluídos participantes que não tenham assinado o Termo de Consentimento Livre e Esclarecido, específico para essa finalidade (TCLE 2 e TCLE 3). Solicita-se adequar a redação, visto que a assinatura do TCLE é condição obrigatória para a inclusão (início) da participação na pesquisa. Não assinar o termo de consentimento é um direito do participante de não participar da pesquisa.

Resposta: A redação foi alterada ao longo do item Critérios de inclusão e exclusão no Projeto detalhado e no campo específico da Plataforma Brasil, conforme solicitado:
¿Todos os participantes serão informados acerca dos procedimentos experimentais e deverão assinar o Termo de Consentimento Livre e Esclarecido, específico para essa finalidade (TCLE 1).¿
¿Todos os participantes serão informados acerca dos procedimentos experimentais e deverão assinar os Termos de Consentimento Livre e Esclarecido, específicos para tais fases (TCLE2 e TCLE3).¿

Vide Arquivo ¿Projeto_detalhado10_12.docx¿ ¿ informação destacada em verde na pág. 56 item 18.

12 - Sobre o deslocamento dos participantes: na fase 1 está previsto deslocamento. Nas demais fases, o participante será avaliado por ocasião do seu atendimento habitual na UBS ou será agendado atendimento com a finalidade de pesquisa? No item 19 consta - As demais fases (diagnóstico), não envolve custos aos participantes ou aos envolvidos no projeto (UFPR ou Prefeituras), dado que tais avaliações constituem ações continuadas de monitoramento dos estados de saúde e de desfechos de fragilidade, fadiga e mobilidade dos idosos. Reitera-se que, caso o deslocamento seja com finalidade de pesquisa, há a necessidade de previsão de ressarcimento ao participante. Solicita-se esclarecer.

Resposta: Acreditamos que a resposta ao item 5 soluciona esse questionamento.

13 - Sobre o uso de dados em nuvem: O projeto prevê¿ o desenvolvimento de uma plataforma computacional composta por um aplicativo para dispositivos móveis, um sistema de informação na Nuvem (sistema Web) com frontend e backend, um sistema de banco de dados e um conjunto de serviços Web a ser disponibilizados via uma API. Desta forma, a arquitetura desta plataforma prevê¿ clientes utilizando aplicativos para dispositivos móveis e frontend do sistema Web conectados e utilizando serviços de um backend em um servidor na Nuvem que oferece uma API de acesso a um serviço de Banco de Dados e outras ações. Solicita-se esclarecer qual será a política de privacidade do aplicativo, e como os dados serão protegidos.

Resposta: Conforme explicitado no projeto detalhado, o acesso à base dos dados coletados dos participantes será restrita aos agentes públicos de saúde que terão acesso controlado por login e senha, a fim de garantir a proteção e confiabilidade dos dados. Os gestores das secretarias municipais de saúde terão acesso ao conjunto de dados agrupados a fim de identificar tendências e executar projeções quanto aos desfechos analisados no estudo, contudo ao agrupar os resultados não é possível identificar os participantes individualmente. Além dos indicativos a serem aportados de forma segmentada, o público em geral poderá ter conhecimento dos resultados do estudo através de um portal que conterá todos os dados agrupados por indicadores de forma simplificada e transparente, sem qualquer possibilidade nesse caso de se identificar os participantes individualmente. Destaca-se que uma das principais finalidades do estudo é justamente fornecer informações tanto aos participantes (via CPF), quanto aos agentes públicos (controlados por níveis de acesso) sobre os estados de saúde para que eles coloquem em andamento medidas preventivas ou corretivas de forma a

sanar os riscos e/ou problemas identificados. Os agentes públicos serão aqueles designados pelas UBSs, submetidos a treinamento específico, os quais já possuem acesso aos prontuários dos usuários. O aplicativo fornecerá aos agentes das UBSs a informação pertinente dos usuários daquela unidade, de maneira a permitir acompanhamento próximo das pessoas atendidas pelo SUS. Finalmente, tecnicamente os dados serão protegidos por ferramentas de segurança de dados contra invasão de hackers e outros tipos de ação cibernética. Os servidores municipais possuem acesso aos prontuários de pacientes do SUS e assume-se que o acesso aos dados do presente estudo não difere daqueles que já são disponibilizados aos servidores. Reitera-se que os presentes testes já são realizados, porém sem sistematização ou análises mais avançadas sobre seus desfechos.
Considerando o exposto, não houve qualquer alteração no texto.

14 -Em relação à propriedade das informações: Nas fases subsequentes (a partir da Fase 2 do estudo) os agentes de saúde das Unidades Básicas de Saúde também terão acesso aos resultados dos testes e poderão entrar em contato para orientar os participantes caso seja necessário. Os resultados de avaliações coletados nas Unidades Básicas de Saúde serão preservados em repositório em nuvem para que possam ser acompanhados longitudinalmente. Ademais, as
avaliações de grandes contingentes permitirão a criação de um banco de dados que poderá prover valores referenciais para que diferentes realidades sejam mais bem compreendidas e abordadas pelo poder público, promovendo a equidade e integralidade na população idosa, conforme um dos objetivos deste estudo. O acesso aos dados da pesquisa deve ser exclusivo aos integrantes da equipe. Dessa forma, solicita-se esclarecer quem serão os agentes de saúde, e se esses serão incluídos (por emenda ao projeto) na Plataforma Brasil e assumirão as responsabilidades descritas em termo próprio. Ademais, solicita-se esclarecer o procedimento de anonimização dos dados desse banco e como serão os procedimentos de manutenção da confidencialidade do estudo.

Resposta: Dado o grande número de agentes de saúde que trabalham nas UBS¿s dos diversos municípios possíveis participantes deste projeto, é inviável inclui-los na Plataforma Brasil por emenda ao projeto. Nesse sentido, salienta-se que apenas os agentes públicos treinados serão habilitados a partir de seu login e senha (possivelmente o mesmo que já possuem para acesso aos demais sistemas utilizados pelas Prefeituras) para ter acesso ao banco de dados a fim de

garantir a confiabilidade dos mesmos. De fato, tais agentes públicos precisam ter acesso aos parâmetros de cada indivíduo a fim de poderem orientar os procedimentos ou intervenções necessárias. De fato, os agentes de saúde das UBSs já possuem acesso aos prontuários dos usuários de forma irrestrita. Logo, não se identifica expressa necessidade em elaborar outros instrumentos que redundam o controle de dados sensíveis. Mais uma vez reitera-se que os testes já são efetuados pelas UBSs, os dados têm a mesma natureza, diferindo apenas na sua forma de obtenção e armazenamento. Logo, os agentes públicos já gozam de acesso a tais dados.
Considerando o exposto, não houve qualquer alteração no texto.

15 - Em relação aos grupos vulneráveis: consta no projeto a informação não se aplica. Solicita-se justificar neste item a inclusão de idosos de 60 a 80 anos, que podem ter autonomia reduzida ou alterações de quadro clínico no decorrer do estudo.

Resposta: Entende-se a observação do parecer. A redação foi ajustada no item 15 do projeto detalhado para acomodar a solicitação, conforme segue:
¿O projeto envolve pessoas idosas que podem ser considerados vulneráveis devido à déficits de mobilidade, perda da autonomia e ou situações de incapacidade física e/ou mental já no início do estudo ou ao longo do tempo tornar-se vulneráveis.¿

Vide Arquivo ¿Projeto_detalhado10_12.docx¿ ¿ informação adicionada e destacada em verde na pág. 55 item 15.

16 - Orçamento: consta o custeio de Frutas e água para os participantes. Quando serão oferecidos aos participantes?

Resposta: As frutas serão ofertadas ao início e final das coletas enquanto a água será ofertada ad libitum. Ressalta-se que tal oferta ocorrerá somente na fase 1, que será realizada nas dependências do Departamento de Educação Física da UFPR. Tal procedimento compreende cortesia aplicada aos estudos pertencentes ao Centro de Estudos do Comportamento Motor, como forma de melhorar o conforto e bem-estar dos participantes.

17 - Solicita-se inserir um orçamento detalhado coerente com os recursos obtidos junto à FINEP, especificando os respectivos gastos e não apenas o que consta de forma bem resumida no projeto.

Resposta: O projeto foi analisado em seu mérito pela FINEP, pela UFPR e pela FUNPAR e considerado como adequado. A análise de mérito do orçamento é objeto de análise de julgamento da proposta.

Destacamos que o projeto possui orçamento para despesas com bolsas, despesas com seguro para os alunos envolvidos, despesas com materiais permanentes, despesas com pessoa jurídica para confecção de aplicativos, despesas com materiais de custeio, despesas com viagens para divulgação dos resultados do estudo, além de viagens nacionais e internacionais para congressos e simpósios. Além disso existe ainda pagamento de despesas de ressarcimento dos custos de gerenciamento à FUNPAR. Para efeitos de transparência e precisão, segue abaixo os prints do orçamento do projeto. Observa-se elevados recursos aportados para bolsas de estudo.

Diante do exposto, as pendências solicitadas foram atendidas.

Considerações Finais a critério do CEP:

01 - Solicitamos que sejam apresentados a este CEP, relatórios semestrais(a cada seis meses de seu parecer de aprovado) e final, sobre o andamento da pesquisa, bem como informações relativas às modificações do protocolo, cancelamento, encerramento e destino dos conhecimentos obtidos, através da Plataforma Brasil - no modo: NOTIFICAÇÃO. Para o próximo relatório, favor utilizar o modelo atualizado, (abril/22), de relatório parcial.

02 - Demais alterações e prorrogação de prazo devem ser enviadas no modo EMENDA. Lembrando que o cronograma de execução da pesquisa deve ser atualizado no sistema Plataforma Brasil antes de enviar solicitação de prorrogação de prazo. Emenda ¿ ver modelo de carta em nossa página: www.cometica.ufpr.br (obrigatório envio).

03 - Importante:(Caso se aplique): Pendências de Coparticipante devem ser respondidas pelo

acesso do Pesquisador principal. Para projetos com coparticipante que também solicitam relatórios semestrais, estes relatórios devem ser enviados por Notificação, pelo login e senha do pesquisador principal no CAAE correspondente a este coparticipante, após o envio do relatório à instituição proponente.

04 ¿ Inserir nos TCLE e TALE o número do CAAE e o número do parecer consubstanciado aprovado, para aplicação dos termos.

Este parecer foi elaborado baseado nos documentos abaixo relacionados:

Tipo Documento	Arquivo	Postagem	Autor	Situação
Informações Básicas do Projeto	PB_INFORMAÇÕES_BÁSICAS_DO_PROJETO_2431843.pdf	11/12/2024 08:19:10		Aceito
Outros	RESPOSTA_ETICA_10_12.docx	10/12/2024 16:56:43	ROBERTA CASTILHOS DETANICO BOHRER	Aceito
Projeto Detalhado / Brochura Investigador	Projeto_detalhado10_12.docx	10/12/2024 16:56:01	ROBERTA CASTILHOS DETANICO BOHRER	Aceito
TCLE / Termos de Assentimento / Justificativa de Ausência	TCLE_3_10_12.docx	10/12/2024 16:55:48	ROBERTA CASTILHOS DETANICO BOHRER	Aceito
TCLE / Termos de Assentimento / Justificativa de Ausência	TCLE_1_10_12.docx	10/12/2024 16:55:35	ROBERTA CASTILHOS DETANICO BOHRER	Aceito
Folha de Rosto	Folha_rosto_ass_05_12.pdf	10/12/2024 16:55:01	ROBERTA CASTILHOS DETANICO BOHRER	Aceito
Outros	Termo_de_imagem.docx	09/12/2024 10:19:11	ROBERTA CASTILHOS DETANICO BOHRER	Aceito
TCLE / Termos de Assentimento / Justificativa de Ausência	TCLE_2_07_10.docx	14/10/2024 13:58:47	ROBERTA CASTILHOS DETANICO BOHRER	Aceito

Outros	Requerimento_apreciacao_projetos.pdf	10/10/2024 11:54:08	ROBERTA CASTILHOS DETANICO BOHRER	Aceito
Outros	Declaracao_ausencia_custos_SMS.pdf	10/10/2024 11:53:32	ROBERTA CASTILHOS DETANICO BOHRER	Aceito
Outros	Carta_Encaminhamento_0710.pdf	10/10/2024 11:52:00	ROBERTA CASTILHOS DETANICO BOHRER	Aceito
Outros	Analise_Merito_ass.pdf	10/10/2024 11:50:31	ROBERTA CASTILHOS DETANICO BOHRER	Aceito
Declaração de concordância	Concordancia_servicos_envolvidos.pdf	10/10/2024 11:50:06	ROBERTA CASTILHOS DETANICO BOHRER	Aceito
Cronograma	Cronograma.pdf	10/10/2024 11:48:51	ROBERTA CASTILHOS DETANICO BOHRER	Aceito
Outros	Checklist.docx	04/10/2024 11:00:19	ROBERTA CASTILHOS DETANICO BOHRER	Aceito
Declaração do Patrocinador	APROVACAO_FINEP.pdf	04/10/2024 10:49:36	ROBERTA CASTILHOS DETANICO BOHRER	Aceito
Orçamento	Orcamento.docx	04/10/2024 10:46:06	ROBERTA CASTILHOS DETANICO BOHRER	Aceito
Outros	APROVACOES.pdf	04/10/2024 10:38:12	ROBERTA CASTILHOS DETANICO BOHRER	Aceito
Outros	Compromisso_EquipePesquisa.pdf	04/10/2024 10:34:46	ROBERTA CASTILHOS DETANICO BOHRER	Aceito
Outros	Carta_SMS_assinada.pdf	03/10/2024 17:31:20	ROBERTA CASTILHOS DETANICO	Aceito

Outros	Carta_SMS_assinada.pdf	03/10/2024 17:31:20	BOHRER	Aceito

Situação do Parecer:
Aprovado

Necessita Apreciação da CONEP:
Não

CURITIBA, 19 de Dezembro de 2024

Assinado por:
IDA CRISTINA GUBERT
(Coordenador(a))
