# Supplementary material for: The influence of arm swing constraint on thigh kinematics, heart rate response, and perceived exertion during the two-minute step test
Source: PLoS One. 2026 Nov 5;21(11):e0361590. doi: 10.1371/journal.pone.0361590 (PMC13739110; doi:10.1371/journal.pone.0361590)
Supplement: Supplementary file 3 [file pone.0361590.s005.docx]

We thank the reviewers for their comments, which have contributed to improving the quality of the manuscript. Below are the point-by-point responses.

General response:

Thank you for raising the question regarding the copyright status of Figure 1. The figure was assembled specifically for this manuscript and has not been reproduced from a previously published scientific article or any other source. Its layout, labels, arrows, smartphone-placement indicators, and depiction of the two experimental conditions were created by the authors (AR). The illustration was modified and incorporated into an original schematic to represent the constrained- and unconstrained-arm-swing conditions evaluated in this study. Accordingly, Figure 1 does not reproduce copyrighted material requiring separate permission from another publisher.

Reviewer #1

| 1. INTRODUCTION  1.1 General: The Introduction is very well done. After reading the abstract, I was unclear on the justification of the study, but the authors did a nice job explaining the scientific foundation for this work. | We thank the reviewer for this positive assessment of the Introduction and for identifying that the study rationale was not sufficiently clear in the Abstract. In response, we comprehensively revised the Abstract to clarify that, although holding a smartphone against the thigh provides a simple means of instrumenting the 2MST, this procedure constrains arm swing and could potentially affect movement, cardiovascular responses, and perceived exertion. We also streamlined the methods and results presented in the Abstract to ensure that the revised version remains within the journal’s 300-word limit. |
| --- | --- |
| 1.2 Lines 97-99: As written, this is an incomplete sentence. It appears combining it with the next sentence would complete the thought, albeit it would make for a quite long sentence. | We agree with the reviewer. The conditional clause was inadvertently separated from its main clause, resulting in an incomplete sentence. Rather than simply combining the two sentences, which would have produced an excessively long sentence, we revised the passage to improve its grammatical structure, clarity, and flow. The revised text reads as follows:  *“If holding the smartphone does not meaningfully alter these outcomes, this would support the use of a smartphone alone—without additional equipment such as straps or a phone pouch—to assess the 2MST.”* |
| 1.3 Line 101: Please add your a priori hypotheses. | We have added our a priori hypotheses at the end of the Introduction. The hypotheses were organized according to the two main outcome domains investigated in the study. Specifically, we hypothesized that constraining arm swing by holding the smartphone against the thigh would not alter: (1) test performance and thigh kinematic outcomes; or (2) heart rate response and perceived exertion.  We also revised the Conclusion section to explicitly indicate the extent to which these hypotheses were supported by the findings. Overall, the hypotheses were largely supported because arm swing constraint did not alter the number of step cycles, mean thigh peak angular velocity, cycle duration, rate of change in thigh peak angular velocity, cycle-duration variability, or heart rate response. However, the variability of thigh peak angular velocity was higher in the constrained condition, and perceived exertion was slightly higher, although the between-condition difference in perceived exertion remained below the minimal detectable change threshold.  The following text was added to the Introduction:  “Our a priori hypotheses were that constraining arm swing by holding the smartphone against the thigh would not alter: (1) test performance and thigh kinematic outcomes, including the number of step cycles, mean thigh peak angular velocity, cycle duration, rate of change in thigh peak angular velocity, and the cycle-to-cycle variability of peak angular velocity and cycle duration; or (2) physiological and perceptual responses, as indicated by heart rate response and perceived exertion.”  The conclusion section was revised as follows:  “The a priori hypotheses were largely supported. Constraining arm swing by holding a smartphone against the thigh did not alter the number of step cycles, mean thigh peak angular velocity, cycle duration, rate of change in thigh peak angular velocity, cycle-duration variability, or heart rate response. However, thigh peak angular velocity variability was higher in the constrained condition, and perceived exertion was slightly higher, although the latter difference remained below the minimal detectable change threshold. These findings indicate that holding a smartphone against the thigh does not meaningfully alter the main outcomes of the 2MST in healthy young adults and support the practical feasibility of performing smartphone-based assessments without additional attachment equipment.” |
| 2. METHODS  2.1 Line 161: What is “11.385 cycles” referring to? Is that the average number of cycles per trial (this seems low) or should the period be a comma to indicate that a total of 11,385 cycles were recorded from all participants/trials? | Thank you for identifying this formatting error. The value refers to the total number of step cycles obtained across all participants and both experimental conditions, rather than the average number of cycles per trial. The period was incorrectly used as the thousands separator and has now been replaced with a comma. We also revised the sentence to improve clarity and correct its grammatical structure.  The revised text reads:  “Then, spatial and temporal variables of the right thigh were determined for each of the 11,385 step cycles using a machine learning–based calibration model.” |
| 2.2 Line 162: Please elaborate on the machine learning-based calibration model developed from the data so future researchers can reproduce your methods. | We agree that the original description was insufficient and that the phrase “developed from the data” was potentially misleading. The machine learning model was not developed or retrained using data from the present study. Instead, we applied a previously developed and validated model that was trained using an independent dataset in which smartphone gyroscope signals were recorded simultaneously with three-dimensional motion capture, which served as the ground-truth reference for thigh peak angular velocity.  We have expanded the 2MST Signal Processing section to summarize the principal preprocessing procedures, predictors, model architecture, and calibration steps. We have also clarified that the development and validation of the machine learning pipeline were not the objective of the present study, which focused on determining the effects of arm swing constraint on thigh kinematics, heart rate response, and perceived exertion. Therefore, the complete model-development, training, validation, and performance procedures remain described in our previous study [25], while the revised manuscript now provides the methodological information necessary to identify and reproduce the pipeline applied here. To accommodate the reviewer’s concern we have included a revised text that reads:  “***Peak thigh angular velocity was estimated for each of the 11,385 step cycles using a previously developed and validated machine learning calibration model [25]. The model was trained using an independent dataset in which motion-capture-derived thigh peak angular velocity served as the supervised learning target. During model development, tri-axial smartphone gyroscope signals were filtered using a zero-lag, second-order low-pass Butterworth filter with a 6 Hz cutoff frequency. Time-domain predictors were extracted from fixed-length windows surrounding each peak and included peak values, selected percentiles, mean, standard deviation, root mean square, signal energy, high-amplitude width, number of zero crossings, cycle duration, and parabolically interpolated peak angular velocity from each gyroscope axis.***  ***The model consisted of a stacked regression framework combining ElasticNet and histogram-based gradient boosting regressors as base learners, with Ridge regression as the meta-learner. The resulting predictions were calibrated using isotonic regression, followed by an additional linear correction applied to the upper 5% of predicted values. In the independent validation dataset, the model presented a mean discrepancy of 1.0°·s⁻¹ relative to motion capture, corresponding to approximately 0.3% of the mean reference value, with limits of agreement ranging from −15.4 to 17.5°·s⁻¹. The previously trained model was applied to the present dataset without retraining. As the development and validation of the machine learning pipeline were not objectives of the present study, which focused on the effects of arm swing constraint, only the principal methodological steps are summarized here. Complete details regarding model development, training, validation, and performance are provided in [25].”*** |
| 3. RESULTS  General: A table showing the data between conditions would be easier to digest than within the text. | We agree that a tabular presentation facilitates a more direct comparison between the experimental conditions. Therefore, we added Table 1 to the Results section, presenting the descriptive statistics for both conditions, together with the corresponding p-values and effect sizes for all kinematic, perceptual, and heart rate outcomes. We also shortened the narrative description of the results section to emphasize the main findings and avoid duplicating all numerical values presented in the table.  “A summary of the descriptive and inferential statistics for all between-condition comparisons is presented in Table 1. Participants performed a comparable number of step cycles in the SW_CON_ and SW_NORM_ conditions. No between-condition differences were observed for mean thigh peak angular velocity, mean cycle duration, rate of change in thigh peak angular velocity, cycle-duration variability, or heart rate response. Variability of thigh peak angular velocity was greater in SW_CON_ than in SW_NORM_. RPE was also slightly higher in SW_CON_; however, the observed difference was below the minimal detectable change.” |
| 4. DISCUSSION  General: The Discussion is well done. The only suggestion I have is for the authors to consider the role of the elastic band that participants were instructed to touch. While the band served as an important way to control for step height, it could also be considered an external focus that may have normalized behavior between conditions unintentionally. The work by Gaby Wulf and colleagues over the years has shown that an external focus of attention has an effect on motor behavior and related constructs. I’m curious if having a consistent external focus during the stepping task may have led to consistent stepping behavior and HR/RPE between conditions. In a real world or clinical setting where the 2MST is deployed, I suspect such an external focus of attention is not there, which could lead to different responses when the arms are constrained vs. not constrained. | We agree that external cues can influence motor behaviour. However, we respectfully clarify that the target used in the present study was not an additional attentional manipulation introduced specifically for the experimental conditions. A predefined knee-elevation height is an inherent component of the standardized 2MST protocol described by Rikli and Jones, in which participants are required to raise the knee to a height corresponding to the midpoint between the patella and the iliac crest [3]. The test therefore cannot be performed with an arbitrary knee-elevation height, as only steps reaching the prescribed target height should be considered as valid.  In clinical and field settings, this target is commonly indicated by a mark or tape placed on a wall or by an equivalent physical or visual reference. In the present study, an elastic band served as a practical alternative to such a marker, allowing the prescribed height to be clearly and consistently identified during both experimental conditions. Others have used comparable approaches (Baghieri et al., 2021 - https://doi.org/10.1016/j.jvn.2021.02.004.) Thus, both conditions were performed with the same standardized height-control procedure, and the elastic band did not selectively favour either condition.  Although we acknowledge that different types of target cues may produce subtly different attentional or sensory effects, examining the influence of visual versus tactile cues was beyond the scope of the present study. More importantly, removing the target altogether would represent a departure from the standardized 2MST protocol rather than a more representative clinical application. Therefore, we have retained the manuscript as originally written. |

Reviewer #2

| 1. The detailed position of the smartphone in two conditions: in line 132 "upper third", line 136 "distal third". Was the smartphone fixed at the same position between the two conditions? If yes, which is the correct position? What was the orientation of the smartphone? If no, why? | The smartphone was positioned at the same location in both experimental conditions, specifically against the anterior aspect of the upper third of the right thigh. The term “distal third” in the description of the SW_CON_ condition was a drafting error and has now been corrected to “proximal third.”  In both conditions, the smartphone was positioned vertically, with the screen facing the participant’s body. The only difference between conditions was the method used to secure the device: in SW_NORM_, the smartphone was secured using an adjustable pouch with elastic straps, whereas in SW_CON_, it was held against the same thigh region by the participant’s right hand. We revised the Experimental Design section to clarify the position and orientation of the smartphone.  “The height of the elastic band was adjusted to the height when standing of the midpoint between the iliac crest and the patella. In the SW_NORM_, a smartphone (iPhone 12, Apple Inc., model A2172, Cupertino, CA, USA) was positioned vertically against the anterior aspect of the proximal third of the right thigh, with the screen facing the participant’s body, and secured using an adjustable pouch with elastic straps to minimize device displacement**.** For this condition, participants were allowed to swing their arms freely without additional instructions, and no control was applied. In the SW_CON_, the phone was held by the participant’s right hand against the same region of the right thigh and maintained in the same vertical orientation, with the screen facing the participant’s body. The opposite hand was positioned over the proximal third of the contralateral thigh at a corresponding height. This meant the swing of both arms was constrained in the SW_CON_ condition. Participants were instructed to maintain the smartphone in this position and minimize its displacement throughout the test. |
| --- | --- |
| 2. How were the data from the gyroscope during walking processed? Even though the reference was cited, the detailed data processing should still be provided. | In response to this concern, as well as a related comment raised by Reviewer 1, we expanded the “2MST Signal Processing” subsection to provide a step-by-step description of the processing applied to the smartphone gyroscope data, from signal extraction and resampling to cycle detection, calibration, and calculation of the kinematic outcomes.  “***Peak thigh angular velocity was estimated for each of the 11,385 step cycles using a previously developed and validated machine learning calibration model [25]. The model was trained using an independent dataset in which motion-capture-derived thigh peak angular velocity served as the supervised learning target. During model development, tri-axial smartphone gyroscope signals were filtered using a zero-lag, second-order low-pass Butterworth filter with a 6 Hz cutoff frequency. Time-domain predictors were extracted from fixed-length windows surrounding each peak and included peak values, selected percentiles, mean, standard deviation, root mean square, signal energy, high-amplitude width, number of zero crossings, cycle duration, and parabolically interpolated peak angular velocity from each gyroscope axis.***  ***The model consisted of a stacked regression framework combining ElasticNet and histogram-based gradient boosting regressors as base learners, with Ridge regression as the meta-learner. The resulting predictions were calibrated using isotonic regression, followed by an additional linear correction applied to the upper 5% of predicted values. In the independent validation dataset, the model presented a mean discrepancy of 1.0°·s⁻¹ relative to motion capture, corresponding to approximately 0.3% of the mean reference value, with limits of agreement ranging from −15.4 to 17.5°·s⁻¹. The previously trained model was applied to the present dataset without retraining. As the development and validation of the machine learning pipeline were not objectives of the present study, which focused on the effects of arm swing constraint, only the principal methodological steps are summarized here. Complete details regarding model development, training, validation, and performance are provided in [25].”*** |
| 3. The rest between two conditions, in line 150, "when heart rate returned to resting HR", the details of the checkpoint should be provided. Was the average heart rate used as a judgment? How long does it take to get the average? | Resting heart rate was defined as the average heart rate recorded during a 10-minute seated resting period performed before the first experimental condition. Following completion of the first 2MST, participants remained seated, and the second condition was initiated only after heart rate had returned to and stabilized around this individual baseline mean.  The inter-condition recovery period lasted approximately five minutes but was extended when necessary, until this criterion was met. We have revised the Experimental Design section to clarify how resting heart rate was determined and used as the recovery checkpoint.  “Before the first experimental condition, resting heart rate was determined as the mean heart rate recorded during a 10-minute seated resting period. Following the first 2MST, participants remained seated for approximately five minutes, or longer, when necessary, until heart rate had returned to and stabilized around their individual resting mean. The second condition was initiated only after this recovery criterion had been met.” |
| 4. In the hand-hold condition, wouldn't it capture more noise movement than the fixed condition, as hand-holding would inevitably create unwanted movement between the hand and the thigh, thus captured by the smartphone? How did your team eliminate the possible accessory movement in the data processing? | We agree that manually holding the smartphone against the thigh may introduce small accessory movements between the device, hand and the thigh. However, no separate artifact-rejection procedure was applied specifically to identify and eliminate these movements.  Instead, their influence was addressed through the machine learning–based calibration model used to estimate thigh peak angular velocity. Importantly, this model was developed and validated in our previous study using data collected under the same hand-held smartphone configuration. Thus, the signals used to train the model already contained the small relative movements, sensor-axis misalignment, and movement artifacts inherent to manually holding the smartphone against the thigh.  The supervised model used tri-axial gyroscope features to learn the relationship between the smartphone signal and thigh angular velocity obtained simultaneously using three-dimensional motion capture as the reference measure. When evaluated using independent unseen data, the machine learning approach showed a bias of only 1.0°·s⁻¹, with limits of agreement ranging from −15.4 to 17.5°·s⁻¹, indicating that accurate thigh angular velocity estimates could be obtained despite the potential accessory movement associated with the hand-held configuration.  Therefore, we do not claim that accessory movement was eliminated; rather, its influence was implicitly incorporated and reduced by the calibration model. The development and validation of this model were not the focus of the present study and are described in detail in our previous publication in Sensors [25]. The present investigation applied this previously validated model to examine whether arm-swing constraint influenced the 2MST outcomes. |
| 5. Discussion of the heart rate, the explanation for the contract paragraph starts at line 272, but is not completed. It ended at the "partially explained". Please elaborate on the possible mechanism. | We have expanded this paragraph to clarify why restricting arm swing may affect heart rate during forward walking but not during marching in place.  We have also clarified that this proposed mechanism remains interpretative because oxygen consumption, centre-of-mass motion, and muscle activation were not directly measured in the present study.  “During forward walking, arm swing counteracts the angular momentum generated by pelvic and trunk rotation and may reduce the muscular effort required to stabilize the trunk and control centre-of-mass motion. Restricting arm swing may therefore require greater compensatory activation of the trunk and lower-limb muscles, increasing metabolic demand and heart rate. In contrast, marching in place involves no forward progression, limited anteroposterior centre-of-mass displacement, and likely less transverse pelvic rotation and whole-body yaw angular momentum. Because body weight is transferred predominantly in the mediolateral direction, the arms may provide less mechanical counterbalance than during forward walking. Consequently, constraining arm swing during the 2MST may require little additional stabilizing work, which could explain the similar heart rate responses between conditions. However, this mechanism remains speculative because centre-of-mass motion, muscle activation, and oxygen consumption were not directly assessed.” |
| 6. In the paragraph started at line 282, comparing the current result of HR and RPE with other populations is not reasonable, as the other populations are mostly not healthy subjects. It is hard to persuade readers to compare populations with diseases with young, healthy subjects. Please explain the purpose of this paragraph. | The original purpose of this paragraph was not to suggest that young healthy participants and clinical populations respond equivalently to the 2MST, but rather to provide descriptive context regarding the relative cardiovascular and perceptual demands elicited by the test.  However, we agree that differences in age, health status, functional capacity, medication use, and study protocols limit the interpretation of comparisons between the present sample and the clinical populations cited. We also acknowledge that the original wording could be interpreted as making a direct physiological comparison between these populations.  Therefore, we removed the cross-population comparison and the associated inference regarding functional capacity. The revised paragraph now focuses exclusively on the within-sample comparison between the constrained and unconstrained arm-swing conditions.  “Within the present sample of young healthy adults, constraining arm swing did not increase the cardiovascular demand of the 2MST. Although perceived exertion was slightly higher in the constrained condition, the magnitude of this difference remained below the minimal detectable change, indicating that it was unlikely to represent a meaningful perceptual alteration.” |
