# Supplementary material for: The influence of arm swing constraint on thigh kinematics, heart rate response, and perceived exertion during the two-minute step test
Source: PLoS One. 2026 Nov 5;21(11):e0361590. doi: 10.1371/journal.pone.0361590 (PMC13739110; doi:10.1371/journal.pone.0361590)
Supplement: Supplementary file 4 [file pone.0361590.s006.docx]

| 1. The expected effect size, the referenced literature used to estimate it, and the detailed input parameters are not specified. It is recommended to provide this missing information to justify the sample size calculation. | We revised the Methods section to detail the parameters considered in the a priori sample size calculation. Because no previous study had directly evaluated the effect of restricting arm motion during the 2-min step test, a directly transferable effect-size estimate based on published data was not available. Therefore, a conventional moderate within-participant effect size of 0.50, as proposed by Cohen (1988)(24), was used to determine the required sample size. The calculation was performed in G*Power version 3.1.9.7 (23) using a t-test for the difference between two dependent means (matched pairs), with a one-tailed hypothesis, α = 0.05, and statistical power of 0.95. These parameters resulted in a minimum required sample size of 45 participants (actual power = 0.951). The corresponding references and detailed input parameters have now been added to the Methods section. |
| --- | --- |
| 2. Regarding the experimental design: The statement "The order of each condition was counterbalanced across the group" lacks necessary methodological detail. Please specify the exact counterbalancing procedure. Specifically, clarify how the 45 participants were allocated to the different testing sequences (e.g., were 22 participants assigned to sequence A-then-B, while 23 were assigned to sequence B-then-A?). A detailed description of this allocation method is required. Furthermore, providing a study flow chart is highly recommended to clearly illustrate the sequence allocation and the overall experimental procedure for readers. | Counterbalancing was performed by successively alternating the testing sequence across participants. Specifically, participants were alternately assigned to the two possible orders (A–B and B–A; A = unrestricted arm motion, B = restricted arm motion), resulting in 23 participants completing the testing sequence in one way and 22 completing it in the reverse sequence. We have revised the Methods section to clarify this procedure, which reads as follows:  “*The order of the two conditions was counterbalanced across participants by successively alternating the testing sequence. Participants were assigned alternately to the two possible testing sequences (A–B and B–A; A = unrestricted arm motion, B = restricted arm motion), resulting in 23 participants completing the testing sequence in one way and 22 completing it in* *the reverse sequence*.” |
| 3. Redundancy (Lines 127-131 and 158-162): The information regarding heart rate in Lines 127-131 is repetitive with the content in Lines 158-162. It is recommended to delete or merge these sections to maintain conciseness. | As suggested, Lines 158-162 have been deleted. |
| 4. Discussion on stepping cycles: In the second paragraph of the Discussion, the number of marching-in-place cycles is compared with previous studies (References 8 and 7). However, Reference 7 focuses on older adults, a population that is not relevant to the current study. It is advised to remove the comparison with Reference 7. | We agree with the reviewer that reference to studies that used older adults is not relevant to the current study. Thus, the comparison made in the text to the number of cycles performed by older adults along with any discussion points referring to older adults have been removed. |
| 5. Limits of Agreement (LoA): The model's limits of agreement, ranging from −15.4 to 17.5°·s⁻¹, appear excessively large for measuring hip sagittal plane kinematics during marching in place or gait cycles. Are there other studies providing comparable data for overground gait, marching in place, or treadmill walking? It is recommended to cite findings from similar literature to justify whether this margin of error is clinically or biomechanically acceptable for ,this context. | We respectfully disagree with the reviewer. The magnitude of the limits of agreement of −15.4 to +17.5°·s⁻¹, should be interpreted relative to the magnitude of the variable being measured (Ludbrook, 2008; Bland & Altman, 1999). In this case, the measure is the thigh’s peak angular velocity, which for the restricted arm motion condition (the condition that recorded the highest angular velocity) a mean value of 328.2 +/- 23.2 °·s⁻¹ was recorded. This means the lower and upper LoA range (~33°·s⁻¹) was within 10% of the measured outcome. Furthermore, since deviations occur in either the positive or negative direction, the expected error in a particular measurement is the upper or lower limit value minus the mean bias, which in this case yields a value of approximately ±16.5°·s⁻¹. Relative to the mean peak angular velocity of 328.2 °·s⁻¹, this corresponds to approximately a **±5% error**, which is not excessively large. |
| 6. Discussion on kinematic variability: The significant difference observed in the variability of peak angular velocity should be thoroughly addressed in the Discussion section. Lines 278-281 are currently brief and could be seamlessly expanded to accommodate this. The authors should discuss whether this increased variability stems from altered movement patterns or increased exertion caused by requiring participants to hold the smartphone themselves. Although this is briefly mentioned in the limitations (Line 343: "constraining the arms and holding the smartphone may introduce changes in the thigh kinematics"), it lacks an in-depth exploration. Furthermore, this increased variability could potentially be an artifact of the model's measurement errors—as mentioned previously, the large limits of agreement might have directly contributed to the high variability in the final outcomes. Both possibilities must be critically discussed. | We agree with the reviewer that further discussion should be included regarding the finding that variability in peak thigh angular velocity was greater for the constrained arm condition compared unconstrained condition. Lines 278-282 have now been expanded to accommodate this additional discussion, as outlined below:  “*T*he results also indicate that although cycle duration variability was similar across conditions, the variability in mean thigh peak angular velocity was greater in the constrained condition compared with the unconstrained condition. This finding agrees with previous reports indicating that constraining arm swing causes greater variability while walking on a treadmill or overground [29,33]. One possible explanation for the greater variability in thigh angular velocity in the constrained condition may relate to changes in angular momentum associated with restricting arm swing. During walking, contralateral arm swing counteracts angular momentum generated by the lower limbs, reducing net whole-body angular momentum about the vertical axis [18]. Thus, constraining arm swing could have affected this process, and the unfamiliarity of this constraint could have contributed to the increased variability in peak thigh angular velocity. Another possible explanation is that requiring participants to hold the smartphone themselves increased exertion, which led to greater variability. In support of this, it is notable that participants’ rating of perceived exertion (RPE) was higher in the constrained condition compared with the unconstrained condition. It is also possible that the act of holding the phone to the thigh increased variability in peak thigh angular velocity by creating greater relative motion between the thigh and the phone (e.g., greater and more variable soft tissue motion) compared with when the phone was strapped to the thigh via the elastic pouch.*”* |
| 7. Reference formatting: There are multiple formatting errors in the reference list, with several citations missing journal names. It is recommended to carefully cross-check and correct the entire reference list to ensure compliance with the journal's guidelines. | We have revised the references and hope that all issues have been resolved. |
| 8. Regarding the conclusion: The authors state that holding a smartphone "does not meaningfully alter the main outcomes of the 2MST... and support[s] the practical feasibility...". This conclusion is not sufficiently conservative and overstates the findings. While the macro task performance (e.g., total step count) might not change significantly, concluding that the unstrapped condition does not "meaningfully alter" the outcomes disregards the crucial finding of a significant increase in the variability of thigh peak angular velocity (effect size = 0.68). In biomechanical and clinical assessments, an altered kinematic variability is indeed a "meaningful" change, as it suggests either a shift in motor control strategies (compensatory movements) or an artifact of increased measurement noise when the device is not firmly attached. Claiming "practical feasibility" without explicitly acknowledging this trade-off in signal quality and movement stability is misleading. The authors must tone down their conclusion and explicitly address the observed kinematic variability and methodological limitations in their final concluding remarks. | We agree with the reviewer and have reduced the assertive tone of the conclusion. The updated text is as follows:  *“However, thigh peak angular velocity variability was higher in the constrained condition, and perceived exertion was slightly elevated, although the latter difference remained below the minimal detectable change threshold.* ***Taken together, these findings suggest that holding a smartphone against the thigh can provide an expedient a*nd *pragmatic means to evaluate the 2MST as the approach doesn’t substantially affect the main outcomes of the 2MST in healthy young adults, and thus they also provide preliminary support for the feasibility of smartphone-based assessments without additional attachment equipment*. *However, given that holding the phone to the thigh did affect the variability in the thigh angular velocity some caution may be needed in using the highlighted approach in all scenarios*.”** |
