# Supplementary material for: The influence of arm swing constraint on thigh kinematics, heart rate response, and perceived exertion during the two-minute step test
Source: PLoS One. 2026 Nov 5;21(11):e0361590. doi: 10.1371/journal.pone.0361590 (PMC13739110; doi:10.1371/journal.pone.0361590)
Supplement: Peer review history

# Peer review: Decision Letter 0

**DOI:** 10.1371/journal.pone.0361590.r001

**Authors:** Anne E. Martin

26 Jul 2026

Dear Dr. Buckley,

Thank you for submitting your manuscript to PLOS ONE. After careful consideration, we feel that it has merit but does not fully meet PLOS ONE’s publication criteria as it currently stands. Therefore, we invite you to submit a revised version of the manuscript that addresses the points raised during the review process.

Please submit your revised manuscript by Sep 24 2026 11:59PM. If you will need more time than this to complete your revisions, please reply to this message or contact the journal office at plosone@plos.org. When you're ready to submit your revision, log on to https://www.editorialmanager.com/pone/ and select the 'Submissions Needing Revision' folder to locate your manuscript file.

- A letter that responds to each point raised by the academic editor and reviewer(s). You should upload this letter as a separate file labeled 'Response to Reviewers'.
- A marked-up copy of your manuscript that highlights changes made to the original version. You should upload this as a separate file labeled 'Revised Manuscript with Track Changes'.
- An unmarked version of your revised paper without tracked changes. You should upload this as a separate file labeled 'Manuscript'.

If you would like to make changes to your financial disclosure, please include your updated statement in your cover letter. Guidelines for resubmitting your figure files are available below the reviewer comments at the end of this letter.

If applicable, we recommend that you deposit your laboratory protocols in protocols.io to enhance the reproducibility of your results. Protocols.io assigns your protocol its own identifier (DOI) so that it can be cited independently in the future. For instructions see: https://journals.plos.org/plosone/s/submission-guidelines#loc-laboratory-protocols. Additionally, PLOS ONE offers an option for publishing peer-reviewed Lab Protocol articles, which describe protocols hosted on protocols.io. Read more information on sharing protocols at https://plos.org/protocols?utm_medium=editorial-email&utm_source=authorletters&utm_campaign=protocols.

As the corresponding author, your ORCID iD is verified in the submission system and will appear in the published article. PLOS supports the use of ORCID, and we encourage all coauthors to register for an ORCID iD and use it as well. Please encourage your coauthors to verify their ORCID iD within the submission system before final acceptance, as unverified ORCID iDs will not appear in the published article. *Only*  the individual author can complete the verification step; PLOS staff *cannot*  verify ORCID iDs on behalf of authors.

We look forward to receiving your revised manuscript.

Kind regards,

Anne E. Martin

Academic Editor

PLOS One

Journal Requirements:

When submitting your revision, we need you to address these additional requirements.

1. Please ensure that your manuscript meets PLOS ONE's style requirements, including those for file naming. The PLOS ONE style templates can be found at

https://journals.plos.org/plosone/s/file?id=wjVg/PLOSOne_formatting_sample_main_body.pdf and https://journals.plos.org/plosone/s/file?id=ba62/PLOSOne_formatting_sample_title_authors_affiliations.pdf

2. Please include a complete copy of PLOS’ questionnaire on inclusivity in global research in your revised manuscript. Our policy for research in this area aims to improve transparency in the reporting of research performed outside of researchers’ own country or community. The policy applies to researchers who have travelled to a different country to conduct research, research with Indigenous populations or their lands, and research on cultural artefacts. The questionnaire can also be requested at the journal’s discretion for any other submissions, even if these conditions are not met.  Please find more information on the policy and a link to download a blank copy of the questionnaire here: https://journals.plos.org/plosone/s/best-practices-in-research-reporting. Please upload a completed version of your questionnaire as Supporting Information when you resubmit your manuscript.

3. We note that Figure 1 in your submission contain copyrighted images. All PLOS content is published under the Creative Commons Attribution License (CC BY 4.0), which means that the manuscript, images, and Supporting Information files will be freely available online, and any third party is permitted to access, download, copy, distribute, and use these materials in any way, even commercially, with proper attribution. For more information, see our copyright guidelines: http://journals.plos.org/plosone/s/licenses-and-copyright.

We require you to either (1) present written permission from the copyright holder to publish these figures specifically under the CC BY 4.0 license, or (2) remove the figures from your submission:

a. You may seek permission from the original copyright holder of Figure 1 to publish the content specifically under the CC BY 4.0 license.

We recommend that you contact the original copyright holder with the Content Permission Form (http://journals.plos.org/plosone/s/file?id=7c09/content-permission-form.pdf) and the following text:

“I request permission for the open-access journal PLOS ONE to publish XXX under the Creative Commons Attribution License (CCAL) CC BY 4.0 (http://creativecommons.org/licenses/by/4.0/). Please be aware that this license allows unrestricted use and distribution, even commercially, by third parties. Please reply and provide explicit written permission to publish XXX under a CC BY license and complete the attached form.”

Please upload the completed Content Permission Form or other proof of granted permissions as an "Other" file with your submission.

In the figure caption of the copyrighted figure, please include the following text: “Reprinted from [ref] under a CC BY license, with permission from [name of publisher], original copyright [original copyright year].”

b. If you are unable to obtain permission from the original copyright holder to publish these figures under the CC BY 4.0 license or if the copyright holder’s requirements are incompatible with the CC BY 4.0 license, please either i) remove the figure or ii) supply a replacement figure that complies with the CC BY 4.0 license. Please check copyright information on all replacement figures and update the figure caption with source information. If applicable, please specify in the figure caption text when a figure is similar but not identical to the original image and is therefore for illustrative purposes only.

4. Please include captions for your Supporting Information files at the end of your manuscript, and update any in-text citations to match accordingly. Please see our Supporting Information guidelines for more information: http://journals.plos.org/plosone/s/supporting-information.

5. We are unable to open your Supporting Information file [renamed_4ff84.zip, DATA.jasp.zip]. Please kindly revise as necessary and re-upload.

6. If the reviewer comments include a recommendation to cite specific previously published works, please review and evaluate these publications to determine whether they are relevant and should be cited. There is no requirement to cite these works unless the editor has indicated otherwise.

[Note: HTML markup is below. Please do not edit.]

Reviewers' comments:

Reviewer's Responses to Questions

**Comments to the Author**

1. Is the manuscript technically sound, and do the data support the conclusions?

Reviewer #1: Yes

Reviewer #2: Partly

**********

2. Has the statistical analysis been performed appropriately and rigorously?

Reviewer #1: Yes

Reviewer #2: Yes

**********

3. Have the authors made all data underlying the findings in their manuscript fully available?

The PLOS Data policy

Reviewer #1: Yes

Reviewer #2: Yes

**********

4. Is the manuscript presented in an intelligible fashion and written in standard English?

Reviewer #1: Yes

Reviewer #2: Yes

**********

Reviewer #1: Comments and suggestions to the authors: This is an interesting paper that will assist with clinical translation. The two-minute step test (2MST) is commonly used to assess mobility and fragility, but its typical deployment relies on subjective assessment. The authors proposed using sensors in a smartphone to objectively measure performance during the 2MST with either the smartphone attached to the leg with a strap or with the participant holding the smartphone against their leg. The latter maximizes clinical translation, as it would afford the ability to deploy the objectively measured task to a broader spectrum of environments. However, holding the smartphone against the leg limits upper limb movement during the task, which could alter task performance, heart rate, and perceived exertion. Exploring these constructs between the two conditions was the focus of this paper, which makes a nice contribution to the literature. Below are a few suggestions to strengthen the clarity and/or interpretation of the results.

INTRODUCTION

General: The Introduction is very well done. After reading the abstract, I was unclear on the justification of the study, but the authors did a nice job explaining the scientific foundation for this work.

Lines 97-99: As written, this is an incomplete sentence. It appears combining it with the next sentence would complete the thought, albeit it would make for a quite long sentence.

Line 101: Please add your a priori hypotheses.

METHODS

Line 161: What is “11.385 cycles” referring to? Is that the average number of cycles per trial (this seems low) or should the period be a comma to indicate that a total of 11,385 cycles were recorded from all participants/trials?

Line 162: Please elaborate on the machine learning-based calibration model developed from the data so future researchers can reproduce your methods.

RESULTS

General: A table showing the data between conditions would be easier to digest than within the text.

DISCUSSION

General: The Discussion is well done. The only suggestion I have is for the authors to consider the role of the elastic band that participants were instructed to touch. While the band served as an important way to control for step height, it could also be considered an external focus that may have normalized behavior between conditions unintentionally. The work by Gaby Wulf and colleagues over the years has shown that an external focus of attention has an effect on motor behavior and related constructs. I’m curious if having a consistent external focus during the stepping task may have led to consistent stepping behavior and HR/RPE between conditions. In a real world or clinical setting where the 2MST is deployed, I suspect such an external focus of attention is not there, which could lead to different responses when the arms are constrained vs. not constrained.

Reviewer #2: The topic of this article is interesting and could provide future studies with a new way to assess walking.

However, some method details are conflicting or lack the detailed explanation required for revision before acceptance.

1. The detailed position of the smartphone in two conditions: in line 132 "upper third", line 136 "distal third". Was the smartphone

fixed at the same position between the two conditions? If yes, which is the correct position? What was the orientation of the smartphone? If no, why?

2. How were the data from the gyroscope during walking processed? Even though the reference was cited, the detailed data processing should still be provided.

3. The rest between two conditions, in line 150, "when heart rate returned to resting HR", the details of the checkpoint should be provided. Was the average heart rate used as a judgment? How long does it take to get the average?

4. In the hand-hold condition, wouldn't it capture more noise movement than the fixed condition, as hand-holding would inevitably create unwanted movement between the hand and the thigh, thus captured by the smartphone? How did your team eliminate the possible accessory movement in the data processing?

5. Discussion of the heart rate, the explanation for the contract paragraph starts at line 272, but is not completed. It ended at the "partially explained". Please elaborate on the possible mechanism.

6. In the paragraph started at line 282, comparing the current result of HR and RPE with other populations is not reasonable, as the other populations are mostly not healthy subjects. It is hard to persuade readers to compare populations with diseases with young, healthy subjects. Please explain the purpose of this paragraph.

**********

what does this mean?). If published, this will include your full peer review and any attached files.

If you choose “no”, your identity will remain anonymous but your review may still be made public.

**Do you want your identity to be public for this peer review?**  For information about this choice, including consent withdrawal, please see our Privacy Policy

Reviewer #1: No

Reviewer #2: No

**********

[NOTE: If reviewer comments were submitted as an attachment file, they will be attached to this email and accessible via the submission site. Please log into your account, locate the manuscript record, and check for the action link "View Attachments". If this link does not appear, there are no attachment files.]

To ensure your figures meet our technical requirements, please review our figure guidelines: https://journals.plos.org/plosone/s/figures

You may also use PLOS’s free figure tool, NAAS, to help you prepare publication quality figures: https://journals.plos.org/plosone/s/figures#loc-tools-for-figure-preparation.

NAAS will assess whether your figures meet our technical requirements by comparing each figure against our figure specifications.

# Author comment: Author response to Decision Letter 1

**DOI:** 10.1371/journal.pone.0361590.r002

13 Aug 2026

We thank the reviewers for their comments, which have contributed to improving the quality of the manuscript. Below are the point-by-point responses.

General response:

Thank you for raising the question regarding the copyright status of Figure 1. The figure was assembled specifically for this manuscript and has not been reproduced from a previously published scientific article or any other source. Its layout, labels, arrows, smartphone-placement indicators, and depiction of the two experimental conditions were created by the authors (AR). The illustration was modified and incorporated into an original schematic to represent the constrained- and unconstrained-arm-swing conditions evaluated in this study. Accordingly, Figure 1 does not reproduce copyrighted material requiring separate permission from another publisher.

Reviewer #1

1.1 General: The Introduction is very well done. After reading the abstract, I was unclear on the justification of the study, but the authors did a nice job explaining the scientific foundation for this work.

Response. We thank the reviewer for this positive assessment of the Introduction and for identifying that the study rationale was not sufficiently clear in the Abstract. In response, we comprehensively revised the Abstract to clarify that, although holding a smartphone against the thigh provides a simple means of instrumenting the 2MST, this procedure constrains arm swing and could potentially affect movement, cardiovascular responses, and perceived exertion. We also streamlined the methods and results presented in the Abstract to ensure that the revised version remains within the journal’s 300-word limit.

2 Lines 97-99: As written, this is an incomplete sentence. It appears combining it with the next sentence would complete the thought, albeit it would make for a quite long sentence.

Response. We agree with the reviewer. The conditional clause was inadvertently separated from its main clause, resulting in an incomplete sentence. Rather than simply combining the two sentences, which would have produced an excessively long sentence, we revised the passage to improve its grammatical structure, clarity, and flow. The revised text reads as follows:

“If holding the smartphone does not meaningfully alter these outcomes, this would support the use of a smartphone alone—without additional equipment such as straps or a phone pouch—to assess the 2MST.”

1.3 Line 101: Please add your a priori hypotheses.

Response. We have added our a priori hypotheses at the end of the Introduction. The hypotheses were organized according to the two main outcome domains investigated in the study. Specifically, we hypothesized that constraining arm swing by holding the smartphone against the thigh would not alter: (1) test performance and thigh kinematic outcomes; or (2) heart rate response and perceived exertion.

We also revised the Conclusion section to explicitly indicate the extent to which these hypotheses were supported by the findings. Overall, the hypotheses were largely supported because arm swing constraint did not alter the number of step cycles, mean thigh peak angular velocity, cycle duration, rate of change in thigh peak angular velocity, cycle-duration variability, or heart rate response. However, the variability of thigh peak angular velocity was higher in the constrained condition, and perceived exertion was slightly higher, although the between-condition difference in perceived exertion remained below the minimal detectable change threshold.

The following text was added to the Introduction:

“Our a priori hypotheses were that constraining arm swing by holding the smartphone against the thigh would not alter: (1) test performance and thigh kinematic outcomes, including the number of step cycles, mean thigh peak angular velocity, cycle duration, rate of change in thigh peak angular velocity, and the cycle-to-cycle variability of peak angular velocity and cycle duration; or (2) physiological and perceptual responses, as indicated by heart rate response and perceived exertion.”

The conclusion section was revised as follows:

“The a priori hypotheses were largely supported. Constraining arm swing by holding a smartphone against the thigh did not alter the number of step cycles, mean thigh peak angular velocity, cycle duration, rate of change in thigh peak angular velocity, cycle-duration variability, or heart rate response. However, thigh peak angular velocity variability was higher in the constrained condition, and perceived exertion was slightly higher, although the latter difference remained below the minimal detectable change threshold. These findings indicate that holding a smartphone against the thigh does not meaningfully alter the main outcomes of the 2MST in healthy young adults and support the practical feasibility of performing smartphone-based assessments without additional attachment equipment.”

2. METHODS

2.1 Line 161: What is “11.385 cycles” referring to? Is that the average number of cycles per trial (this seems low) or should the period be a comma to indicate that a total of 11,385 cycles were recorded from all participants/trials?

Response. Thank you for identifying this formatting error. The value refers to the total number of step cycles obtained across all participants and both experimental conditions, rather than the average number of cycles per trial. The period was incorrectly used as the thousands separator and has now been replaced with a comma. We also revised the sentence to improve clarity and correct its grammatical structure.

The revised text reads:

“Then, spatial and temporal variables of the right thigh were determined for each of the 11,385 step cycles using a machine learning–based calibration model.”

2.2 Line 162: Please elaborate on the machine learning-based calibration model developed from the data so future researchers can reproduce your methods.

Response. We agree that the original description was insufficient and that the phrase “developed from the data” was potentially misleading. The machine learning model was not developed or retrained using data from the present study. Instead, we applied a previously developed and validated model that was trained using an independent dataset in which smartphone gyroscope signals were recorded simultaneously with three-dimensional motion capture, which served as the ground-truth reference for thigh peak angular velocity.

We have expanded the 2MST Signal Processing section to summarize the principal preprocessing procedures, predictors, model architecture, and calibration steps. We have also clarified that the development and validation of the machine learning pipeline were not the objective of the present study, which focused on determining the effects of arm swing constraint on thigh kinematics, heart rate response, and perceived exertion. Therefore, the complete model-development, training, validation, and performance procedures remain described in our previous study [25], while the revised manuscript now provides the methodological information necessary to identify and reproduce the pipeline applied here. To accommodate the reviewer’s concern we have included a revised text that reads:

“Peak thigh angular velocity was estimated for each of the 11,385 step cycles using a previously developed and validated machine learning calibration model [25]. The model was trained using an independent dataset in which motion-capture-derived thigh peak angular velocity served as the supervised learning target. During model development, tri-axial smartphone gyroscope signals were filtered using a zero-lag, second-order low-pass Butterworth filter with a 6 Hz cutoff frequency. Time-domain predictors were extracted from fixed-length windows surrounding each peak and included peak values, selected percentiles, mean, standard deviation, root mean square, signal energy, high-amplitude width, number of zero crossings, cycle duration, and parabolically interpolated peak angular velocity from each gyroscope axis.

The model consisted of a stacked regression framework combining ElasticNet and histogram-based gradient boosting regressors as base learners, with Ridge regression as the meta-learner. The resulting predictions were calibrated using isotonic regression, followed by an additional linear correction applied to the upper 5% of predicted values. In the independent validation dataset, the model presented a mean discrepancy of 1.0°·s⁻¹ relative to motion capture, corresponding to approximately 0.3% of the mean reference value, with limits of agreement ranging from −15.4 to 17.5°·s⁻¹. The previously trained model was applied to the present dataset without retraining. As the development and validation of the machine learning pipeline were not objectives of the present study, which focused on the effects of arm swing constraint, only the principal methodological steps are summarized here. Complete details regarding model development, training, validation, and performance are provided in [25].”

3. RESULTS

General: A table showing the data between conditions would be easier to digest than within the text.

Response. We agree that a tabular presentation facilitates a more direct comparison between the experimental conditions. Therefore, we added Table 1 to the Results section, presenting the descriptive statistics for both conditions, together with the corresponding p-values and effect sizes for all kinematic, perceptual, and heart rate outcomes. We also shortened the narrative description of the results section to emphasize the main findings and avoid duplicating all numerical values presented in the table.

“A summary of the descriptive and inferential statistics for all between-condition comparisons is presented in Table 1. Participants performed a comparable number of step cycles in the SWCON and SWNORM conditions. No between-condition differences were observed for mean thigh peak angular velocity, mean cycle duration, rate of change in thigh peak angular velocity, cycle-duration variability, or heart rate response. Variability of thigh peak angular velocity was greater in SWCON than in SWNORM. RPE was also slightly higher in SWCON; however, the observed difference was below the minimal detectable change.”

4. DISCUSSION

General: The Discussion is well done. The only suggestion I have is for the authors to consider the role of the elastic band that participants were instructed to touch. While the band served as an important way to control for step height, it could also be considered an external focus that may have normalized behavior between conditions unintentionally. The work by Gaby Wulf and colleagues over the years has shown that an external focus of attention has an effect on motor behavior and related constructs. I’m curious if having a consistent external focus during the stepping task may have led to consistent stepping behavior and HR/RPE between conditions. In a real world or clinical setting where the 2MST is deployed, I suspect such an external focus of attention is not there, which could lead to different responses when the arms are constrained vs. not constrained.

Response. We agree that external cues can influence motor behaviour. However, we respectfully clarify that the target used in the present study was not an additional attentional manipulation introduced specifically for the experimental conditions. A predefined knee-elevation height is an inherent component of the standardized 2MST protocol described by Rikli and Jones, in which participants are required to raise the knee to a height corresponding to the midpoint between the patella and the iliac crest [3]. The test therefore cannot be performed with an arbitrary knee-elevation height, as only steps reaching the prescribed target height should be considered as valid.

In clinical and field settings, this target is commonly indicated by a mark or tape placed on a wall or by an equivalent physical or visual reference. In the present study, an elastic band served as a practical alternative to such a marker, allowing the prescribed height to be clearly and consistently identified during both experimental conditions. Others have used comparable approaches (Baghieri et al., 2021 - https://doi.org/10.1016/j.jvn.2021.02.004.) Thus, both conditions were performed with the same standardized height-control procedure, and the elastic band did not selectively favour either condition.

Although we acknowledge that different types of target cues may produce subtly different attentional or sensory effects, examining the influence of visual versus tactile cues was beyond the scope of the present study. More importantly, removing the target altogether would represent a departure from the standardized 2MST protocol rather than a more representative clinical application. Therefore, we have retained the manuscript as originally written.

Reviewer #2

1. The detailed position of the smartphone in two conditions: in line 132 "upper third", line 136 "distal third". Was the smartphone

fixed at the same position between the two conditions? If yes, which is the correct position? What was the orientation of the smartphone? If no, why?

Response. The smartphone was positioned at the same location in both experimental conditions, specifically against the anterior aspect of the upper third of the right thigh. The term “distal third” in the description of the SWCON condition was a drafting error and has now been corrected to “proximal third.”

In both conditions, the smartphone was positioned vertically, with the screen facing the participant’s body. The only difference between conditions was the method used to secure the device: in SWNORM, the smartphone was secured using an adjustable pouch with elastic straps, whereas in SWCON, it was held against the same thigh region by the participant’s right hand. We revised the Experimental Design section to clarify the position and orientation of the smartphone.

“The height of the elastic band was adjusted to the height when standing of the midpoint between the iliac crest and the patella. In the SWNORM, a smartphone (iPhone 12, Apple Inc., model A2172, Cupertino, CA, USA) was positioned vertically against the anterior aspect of the proximal third of the right thigh, with the screen facing the participant’s body, and secured using an adjustable pouch with elastic straps to minimize device displacement. For this condition, participants were allowed to swing their arms freely without additional instructions, and no control was applied. In the SWCON, the phone was held by the participant’s right hand against the same region of the right thigh and maintained in the same vertical orientation, with the screen facing the participant’s body. The opposite hand was positioned over the proximal third of the contralateral thigh at a corresponding height. This meant the swing of both arms was constrained in the SWCON condition. Participants were instructed to maintain the smartphone in this position and minimize its displacement throughout the test.

2. How were the data from the gyroscope during walking processed? Even though the reference was cited, the detailed data processing should still be provided.

Response. In response to this concern, as well as a related comment raised by Reviewer 1, we expanded the “2MST Signal Processing” subsection to provide a step-by-step description of the processing applied to the smartphone gyroscope data, from signal extraction and resampling to cycle detection, calibration, and calculation of the kinematic outcomes.

“Peak thigh angular velocity was estimated for each of the 11,385 step cycles using a previously developed and validated machine learning calibration model [25]. The model was trained using an independent dataset in which motion-capture-derived thigh peak angular velocity served as the supervised learning target. During model development, tri-axial smartphone gyroscope signals were filtered using a zero-lag, second-order low-pass Butterworth filter with a 6 Hz cutoff frequency. Time-domain predictors were extracted from fixed-length windows surrounding each peak and included peak values, selected percentiles, mean, standard deviation, root mean square, signal energy, high-amplitude width, number of zero crossings, cycle duration, and parabolically interpolated peak angular velocity from each gyroscope axis.

The model consisted of a stacked regression framework combining ElasticNet and histogram-based gradient boosting regressors as base learners, with Ridge regression as the meta-learner. The resulting predictions were calibrated using isotonic regression, followed by an additional linear correction applied to the upper 5% of predicted values. In the independent validation dataset, the model presented a mean discrepancy of 1.0°·s⁻¹ relative to motion capture, corresponding to approximately 0.3% of the mean reference value, with limits of agreement ranging from −15.4 to 17.5°·s⁻¹. The previously trained model was applied to the present dataset without retraining. As the development and validation of the machine learning pipeline were not objectives of the present study, which focused on the effects of arm swing constraint, only the principal methodological steps are summarized here. Complete details regarding model development, training, validation, and performance are provided in [25].”

3. The rest between two conditions, in line 150, "when heart rate returned to resting HR", the details of the checkpoint should be provided. Was the average heart rate used as a judgment? How long does it take to get the average?

Response. Resting heart rate was defined as the average heart rate recorded during a 10-minute seated resting period performed before the first experimental condition. Following completion of the first 2MST, participants remained seated, and the second condition was initiated only after heart rate had returned to and stabilized around this individual baseline mean.

The inter-condition recovery period lasted approximately five minutes but was extended when necessary, until this criterion was met. We have revised the Experimental Design section to clarify how resting heart rate was determined and used as the recovery checkpoint.

“Before the first experimental condition, resting heart rate was determined as the mean heart rate recorded during a 10-minute seated resting period. Following the first 2MST, participants remained seated for approximately five minutes, or longer, when necessary, until heart rate had returned to and stabilized around their individual resting mean. The second condition was initiated only after this recovery criterion had been met.”

4. In the hand-hold condition, wouldn't it capture more noise movement than the fixed condition, as hand-holding would inevitably create unwanted movement between the hand and the thigh, thus captured by the smartphone? How did your team eliminate the possible accessory movement in the data processing?

Response. We agree that manually holding the smartphone against the thigh may introduce small accessory movements between the device, hand and the thigh. However, no separate artifact-rejection procedure was applied specifically to identify and eliminate these movements.

Instead, their influence was addressed through the machine learning–based calibration model used to estimate thigh peak angular velocity. Importantly, this model was developed and validated in our previous study using data collected under the same hand-held smartphone configuration. Thus, the signals used to train the model already contained the small relative movements, sensor-axis misalignment, and movement artifacts inherent to manually holding the smartphone against the thigh.

The supervised model used tri-axial gyroscope features to learn the relationship between the smartphone signal and thigh angular velocity obtained simultaneously using three-dimensional motion capture as the reference measure. When evaluated using independent unseen data, the machine learning approach showed a bias of only 1.0°·s⁻¹, with limits of agreement ranging from −15.4 to 17.5°·s⁻¹, indicating that accurate thigh angular velocity estimates could be obtained despite the potential accessory movement associated with the hand-held configuration.

Therefore, we do not claim that accessory movement was eliminated; rather, its influence was implicitly incorporated and reduced by the calibration model. The development and validation of this model were not the focus of the present study and are described in detail in our previous publication in Sensors [25]. The present investigation applied this previously validated model to examine whether arm-swing constraint influenced the 2MST outcomes.

5. Discussion of the heart rate, the explanation for the contract paragraph starts at line 272, but is not completed. It ended at the "partially explained". Please elaborate on the possible mechanism.

Response. We have expanded this paragraph to clarify why restricting arm swing may affect heart rate during forward walking but not during marching in place.

We have also clarified that this proposed mechanism remains interpretative because oxygen consumption, centre-of-mass motion, and muscle activation were not directly measured in the present study.

“During forward walking, arm swing counteracts the angular momentum generated by pelvic and trunk rotation and may reduce the muscular effort required to stabilize the trunk and control centre-of-mass motion. Restricting arm swing may therefore require greater compensatory activation of the trunk and lower-limb muscles, increasing metabolic demand and heart rate. In contrast, marching in place involves no forward progression, limited anteroposterior centre-of-mass displacement, and likely less transverse pelvic rotation and whole-body yaw angular momentum. Because body weight is transferred predominantly in the mediolateral direction, the arms may provide less mechanical counterbalance than during forward walking. Consequently, constraining arm swing during the 2MST may require little additional stabilizing work, which could explain the similar heart rate responses between conditions. However, this mechanism remains speculative because centre-of-mass motion, muscle activation, and oxygen consumption were not directly assessed.”

6. In the paragraph started at line 282, comparing the current result of HR and RPE with other populations is not reasonable, as the other populations are mostly not healthy subjects. It is hard to persuade readers to compare populations with diseases with young, healthy subjects. Please explain the purpose of this paragraph.

Response. The original purpose of this paragraph was not to suggest that young healthy participants and clinical populations respond equivalently to the 2MST, but rather to provide descriptive context regarding the relative cardiovascular and perceptual demands elicited by the test.

However, we agree that differences in age, health status, functional capacity, medication use, and study protocols limit the interpretation of comparisons between the present sample and the clinical populations cited. We also acknowledge that the original wording could be interpreted as making a direct physiological comparison between these populations.

Therefore, we removed the cross-population comparison and the associated inference regarding functional capacity. The revised paragraph now focuses exclusively on the within-sample comparison between the constrained and unconstrained arm-swing conditions.

“Within the present sample of young healthy adults, constraining arm swing did not increase the cardiovascular demand of the 2MST. Although perceived exertion was slightly higher in the constrained condition, the magnitude of this difference remained below the minimal detectable change, indicating that it was unlikely to represent a meaningful perceptual alteration.”

Attachment Submitted filename: RESPONSES_Plos_28.07.docx

# Peer review: Decision Letter 1

**DOI:** 10.1371/journal.pone.0361590.r003

**Authors:** Anne E. Martin

6 Oct 2026

Dear Dr. Buckley,

Thank you for submitting your manuscript to PLOS One. After careful consideration, we feel that it has merit but does not fully meet PLOS One’s publication criteria as it currently stands. Therefore, we invite you to submit a revised version of the manuscript that addresses the points raised during the review process.

Please submit your revised manuscript by Dec 05 2026 11:59PM. If you will need more time than this to complete your revisions, please reply to this message or contact the journal office at plosone@plos.org. When you're ready to submit your revision, log on to https://www.editorialmanager.com/pone/ and select the 'Submissions Needing Revision' folder to locate your manuscript file.

- A letter that responds to each point raised by the academic editor and reviewer(s). You should upload this letter as a separate file labeled 'Response to Reviewers'.
- A marked-up copy of your manuscript that highlights changes made to the original version. You should upload this as a separate file labeled 'Revised Manuscript with Track Changes'.
- An unmarked version of your revised paper without tracked changes. You should upload this as a separate file labeled 'Manuscript'.

If you would like to make changes to your financial disclosure, please include your updated statement in your cover letter. Guidelines for resubmitting your figure files are available below the reviewer comments at the end of this letter.

If applicable, we recommend that you deposit your laboratory protocols in protocols.io to enhance the reproducibility of your results. Protocols.io assigns your protocol its own identifier (DOI) so that it can be cited independently in the future. For instructions see: https://journals.plos.org/plosone/s/submission-guidelines#loc-laboratory-protocols. Additionally, PLOS One offers an option for publishing peer-reviewed Lab Protocol articles, which describe protocols hosted on protocols.io. Read more information on sharing protocols at https://plos.org/protocols?utm_medium=editorial-email&utm_source=authorletters&utm_campaign=protocols.

As the corresponding author, your ORCID iD is verified in the submission system and will appear in the published article. PLOS supports the use of ORCID, and we encourage all coauthors to register for an ORCID iD and use it as well. Please encourage your coauthors to verify their ORCID iD within the submission system before final acceptance, as unverified ORCID iDs will not appear in the published article. *Only*  the individual author can complete the verification step; PLOS staff *cannot*  verify ORCID iDs on behalf of authors.

We look forward to receiving your revised manuscript.

Kind regards,

Anne E. Martin

Academic Editor

PLOS One

Journal Requirements:

1. If the reviewer comments include a recommendation to cite specific previously published works, please review and evaluate these publications to determine whether they are relevant and should be cited. There is no requirement to cite these works unless the editor has indicated otherwise.

Additional Editor Comments:

While Reviewer 1 is satisfied with your revisions, Reviewer 2 has identified several additional places where clarification or revisions are needed.

[Note: HTML markup is below. Please do not edit.]

Reviewer's Responses to Questions

**Comments to the Author**

Reviewer #1: All comments have been addressed

Reviewer #2: All comments have been addressed

**********

2. Is the manuscript technically sound, and do the data support the conclusions?

Reviewer #1: Yes

Reviewer #2: Partly

**********

3. Has the statistical analysis been performed appropriately and rigorously?

Reviewer #1: Yes

Reviewer #2: Yes

**********

4. Have the authors made all data underlying the findings in their manuscript fully available?

The PLOS Data policy

Reviewer #1: Yes

Reviewer #2: Yes

**********

5. Is the manuscript presented in an intelligible fashion and written in standard English?

Reviewer #1: Yes

Reviewer #2: Yes

**********

Reviewer #1: The authors have done a good job addressing my previous comments. I recommend this paper for publication in its current format.

Reviewer #2: 1. Regarding the sample size analysis: The expected effect size, the referenced literature used to estimate it, and the detailed input parameters are not specified. It is recommended to provide this missing information to justify the sample size calculation.

2. Regarding the experimental design: The statement "The order of each condition was counterbalanced across the group" lacks necessary methodological detail. Please specify the exact counterbalancing procedure. Specifically, clarify how the 45 participants were allocated to the different testing sequences (e.g., were 22 participants assigned to sequence A-then-B, while 23 were assigned to sequence B-then-A?). A detailed description of this allocation method is required. Furthermore, providing a study flow chart is highly recommended to clearly illustrate the sequence allocation and the overall experimental procedure for readers.

3. Redundancy (Lines 127-131 and 158-162): The information regarding heart rate in Lines 127-131 is repetitive with the content in Lines 158-162. It is recommended to delete or merge these sections to maintain conciseness.

4. Discussion on stepping cycles: In the second paragraph of the Discussion, the number of marching-in-place cycles is compared with previous studies (References 8 and 7). However, Reference 7 focuses on older adults, a population that is not relevant to the current study. It is advised to remove the comparison with Reference 7.

5. Limits of Agreement (LoA): The model's limits of agreement, ranging from −15.4 to 17.5°·s⁻¹, appear excessively large for measuring hip sagittal plane kinematics during marching in place or gait cycles. Are there other studies providing comparable data for overground gait, marching in place, or treadmill walking? It is recommended to cite findings from similar literature to justify whether this margin of error is clinically or biomechanically acceptable for this context.

6. Discussion on kinematic variability: The significant difference observed in the variability of peak angular velocity should be thoroughly addressed in the Discussion section. Lines 278-281 are currently brief and could be seamlessly expanded to accommodate this. The authors should discuss whether this increased variability stems from altered movement patterns or increased exertion caused by requiring participants to hold the smartphone themselves. Although this is briefly mentioned in the limitations (Line 343: "constraining the arms and holding the smartphone may introduce changes in the thigh kinematics"), it lacks an in-depth exploration. Furthermore, this increased variability could potentially be an artifact of the model's measurement errors—as mentioned previously, the large limits of agreement might have directly contributed to the high variability in the final outcomes. Both possibilities must be critically discussed.

7. Reference formatting: There are multiple formatting errors in the reference list, with several citations missing journal names. It is recommended to carefully cross-check and correct the entire reference list to ensure compliance with the journal's guidelines.

8. Regarding the conclusion: The authors state that holding a smartphone "does not meaningfully alter the main outcomes of the 2MST... and support[s] the practical feasibility...". This conclusion is not sufficiently conservative and overstates the findings. While the macro task performance (e.g., total step count) might not change significantly, concluding that the unstrapped condition does not "meaningfully alter" the outcomes disregards the crucial finding of a significant increase in the variability of thigh peak angular velocity (effect size = 0.68). In biomechanical and clinical assessments, an altered kinematic variability is indeed a "meaningful" change, as it suggests either a shift in motor control strategies (compensatory movements) or an artifact of increased measurement noise when the device is not firmly attached. Claiming "practical feasibility" without explicitly acknowledging this trade-off in signal quality and movement stability is misleading. The authors must tone down their conclusion and explicitly address the observed kinematic variability and methodological limitations in their final concluding remarks.

**********

what does this mean?). If published, this will include your full peer review and any attached files.

If you choose “no”, your identity will remain anonymous but your review may still be made public.

**Do you want your identity to be public for this peer review?**  For information about this choice, including consent withdrawal, please see our Privacy Policy

Reviewer #1: No

Reviewer #2: No

**********

[NOTE: If reviewer comments were submitted as an attachment file, they will be attached to this email and accessible via the submission site. Please log into your account, locate the manuscript record, and check for the action link "View Attachments". If this link does not appear, there are no attachment files.]

To ensure your figures meet our technical requirements, please review our figure guidelines: https://journals.plos.org/plosone/s/figures

You may also use PLOS’s free figure tool, NAAS, to help you prepare publication quality figures: https://journals.plos.org/plosone/s/figures#loc-tools-for-figure-preparation.

NAAS will assess whether your figures meet our technical requirements by comparing each figure against our figure specifications.

# Author comment: Author response to Decision Letter 2

**DOI:** 10.1371/journal.pone.0361590.r004

9 Oct 2026

1. The expected effect size, the referenced literature used to estimate it, and the detailed input parameters are not specified. It is recommended to provide this missing information to justify the sample size calculation.

Response. We revised the Methods section to detail the parameters considered in the a priori sample size calculation. Because no previous study had directly evaluated the effect of restricting arm motion during the 2-min step test, a directly transferable effect-size estimate based on published data was not available. Therefore, a conventional moderate within-participant effect size of 0.50, as proposed by Cohen (1988)(24), was used to determine the required sample size. The calculation was performed in G*Power version 3.1.9.7 (23) using a t-test for the difference between two dependent means (matched pairs), with a one-tailed hypothesis, α = 0.05, and statistical power of 0.95. These parameters resulted in a minimum required sample size of 45 participants (actual power = 0.951). The corresponding references and detailed input parameters have now been added to the Methods section.

2. Regarding the experimental design: The statement "The order of each condition was counterbalanced across the group" lacks necessary methodological detail. Please specify the exact counterbalancing procedure. Specifically, clarify how the 45 participants were allocated to the different testing sequences (e.g., were 22 participants assigned to sequence A-then-B, while 23 were assigned to sequence B-then-A?). A detailed description of this allocation method is required. Furthermore, providing a study flow chart is highly recommended to clearly illustrate the sequence allocation and the overall experimental procedure for readers.

Response. Counterbalancing was performed by successively alternating the testing sequence across participants. Specifically, participants were alternately assigned to the two possible orders (A–B and B–A; A = unrestricted arm motion, B = restricted arm motion), resulting in 23 participants completing the testing sequence in one way and 22 completing it in the reverse sequence. We have revised the Methods section to clarify this procedure, which reads as follows:

“The order of the two conditions was counterbalanced across participants by successively alternating the testing sequence. Participants were assigned alternately to the two possible testing sequences (A–B and B–A; A = unrestricted arm motion, B = restricted arm motion), resulting in 23 participants completing the testing sequence in one way and 22 completing it in the reverse sequence.”

3. Redundancy (Lines 127-131 and 158-162): The information regarding heart rate in Lines 127-131 is repetitive with the content in Lines 158-162. It is recommended to delete or merge these sections to maintain conciseness.

Response. As suggested, Lines 158-162 have been deleted.

4. Discussion on stepping cycles: In the second paragraph of the Discussion, the number of marching-in-place cycles is compared with previous studies (References 8 and 7). However, Reference 7 focuses on older adults, a population that is not relevant to the current study. It is advised to remove the comparison with Reference 7.

Response. We agree with the reviewer that reference to studies that used older adults is not relevant to the current study. Thus, the comparison made in the text to the number of cycles performed by older adults along with any discussion points referring to older adults have been removed.

5. Limits of Agreement (LoA): The model's limits of agreement, ranging from −15.4 to 17.5°·s⁻¹, appear excessively large for measuring hip sagittal plane kinematics during marching in place or gait cycles. Are there other studies providing comparable data for overground gait, marching in place, or treadmill walking? It is recommended to cite findings from similar literature to justify whether this margin of error is clinically or biomechanically acceptable for ,this context.

Response. We respectfully disagree with the reviewer. The magnitude of the limits of agreement of −15.4 to +17.5°·s⁻¹, should be interpreted relative to the magnitude of the variable being measured (Ludbrook, 2008; Bland & Altman, 1999). In this case, the measure is the thigh’s peak angular velocity, which for the restricted arm motion condition (the condition that recorded the highest angular velocity) a mean value of 328.2 +/- 23.2 °·s⁻¹ was recorded. This means the lower and upper LoA range (~33°·s⁻¹) was within 10% of the measured outcome. Furthermore, since deviations occur in either the positive or negative direction, the expected error in a particular measurement is the upper or lower limit value minus the mean bias, which in this case yields a value of approximately ±16.5°·s⁻¹. Relative to the mean peak angular velocity of 328.2 °·s⁻¹, this corresponds to approximately a ±5% error, which is not excessively large.

6. Discussion on kinematic variability: The significant difference observed in the variability of peak angular velocity should be thoroughly addressed in the Discussion section. Lines 278-281 are currently brief and could be seamlessly expanded to accommodate this. The authors should discuss whether this increased variability stems from altered movement patterns or increased exertion caused by requiring participants to hold the smartphone themselves. Although this is briefly mentioned in the limitations (Line 343: "constraining the arms and holding the smartphone may introduce changes in the thigh kinematics"), it lacks an in-depth exploration. Furthermore, this increased variability could potentially be an artifact of the model's measurement errors—as mentioned previously, the large limits of agreement might have directly contributed to the high variability in the final outcomes. Both possibilities must be critically discussed.

Response. We agree with the reviewer that further discussion should be included regarding the finding that variability in peak thigh angular velocity was greater for the constrained arm condition compared unconstrained condition. Lines 278-282 have now been expanded to accommodate this additional discussion, as outlined below:

“The results also indicate that although cycle duration variability was similar across conditions, the variability in mean thigh peak angular velocity was greater in the constrained condition compared with the unconstrained condition. This finding agrees with previous reports indicating that constraining arm swing causes greater variability while walking on a treadmill or overground [29,33]. One possible explanation for the greater variability in thigh angular velocity in the constrained condition may relate to changes in angular momentum associated with restricting arm swing. During walking, contralateral arm swing counteracts angular momentum generated by the lower limbs, reducing net whole-body angular momentum about the vertical axis [18]. Thus, constraining arm swing could have affected this process, and the unfamiliarity of this constraint could have contributed to the increased variability in peak thigh angular velocity. Another possible explanation is that requiring participants to hold the smartphone themselves increased exertion, which led to greater variability. In support of this, it is notable that participants’ rating of perceived exertion (RPE) was higher in the constrained condition compared with the unconstrained condition. It is also possible that the act of holding the phone to the thigh increased variability in peak thigh angular velocity by creating greater relative motion between the thigh and the phone (e.g., greater and more variable soft tissue motion) compared with when the phone was strapped to the thigh via the elastic pouch.”

7. Reference formatting: There are multiple formatting errors in the reference list, with several citations missing journal names. It is recommended to carefully cross-check and correct the entire reference list to ensure compliance with the journal's guidelines.

Response. We have revised the references and hope that all issues have been resolved.

8. Regarding the conclusion: The authors state that holding a smartphone "does not meaningfully alter the main outcomes of the 2MST... and support[s] the practical feasibility...". This conclusion is not sufficiently conservative and overstates the findings. While the macro task performance (e.g., total step count) might not change significantly, concluding that the unstrapped condition does not "meaningfully alter" the outcomes disregards the crucial finding of a significant increase in the variability of thigh peak angular velocity (effect size = 0.68). In biomechanical and clinical assessments, an altered kinematic variability is indeed a "meaningful" change, as it suggests either a shift in motor control strategies (compensatory movements) or an artifact of increased measurement noise when the device is not firmly attached. Claiming "practical feasibility" without explicitly acknowledging this trade-off in signal quality and movement stability is misleading. The authors must tone down their conclusion and explicitly address the observed kinematic variability and methodological limitations in their final concluding remarks.

Response. We agree with the reviewer and have reduced the assertive tone of the conclusion. The updated text is as follows:

“However, thigh peak angular velocity variability was higher in the constrained condition, and perceived exertion was slightly elevated, although the latter difference remained below the minimal detectable change threshold. Taken together, these findings suggest that holding a smartphone against the thigh can provide an expedient and pragmatic means to evaluate the 2MST as the approach doesn’t substantially affect the main outcomes of the 2MST in healthy young adults, and thus they also provide preliminary support for the feasibility of smartphone-based assessments without additional attachment equipment. However, given that holding the phone to the thigh did affect the variability in the thigh angular velocity some caution may be needed in using the highlighted approach in all scenarios.”

Attachment Submitted filename: Response to Reviewers R2.docx

# Editor report: Decision Letter 2

**DOI:** 10.1371/journal.pone.0361590.r005

**Authors:** Anne E. Martin

14 Oct 2026

The Influence of Arm Swing Constraint on Thigh Kinematics, Heart Rate Response, and Perceived Exertion During the Two-Minute Step Test.

PONE-D-26-26610R2

Dear Dr. Buckley,

We’re pleased to inform you that your manuscript has been judged scientifically suitable for publication and will be formally accepted for publication once it meets all outstanding technical requirements.

Within one week, you’ll receive an e-mail detailing the required amendments. When these have been addressed, you’ll receive a formal acceptance letter and your manuscript will be scheduled for publication.

An invoice will be generated when your article is formally accepted. Please note, if your institution has a publishing partnership with PLOS and your article meets the relevant criteria, all or part of your publication costs will be covered. Please make sure your user information is up-to-date by logging into Editorial Manager at Editorial Manager® and clicking the ‘Update My Information' link at the top of the page. For questions related to billing, please contact billing support.

If your institution or institutions have a press office, please notify them about your upcoming paper to help maximize its impact. If they’ll be preparing press materials, please inform our press team as soon as possible -- no later than 48 hours after receiving the formal acceptance. Your manuscript will remain under strict press embargo until 2 pm Eastern Time on the date of publication. For more information, please contact onepress@plos.org.

Kind regards,

Anne E. Martin

Academic Editor

PLOS One

Additional Editor Comments (optional):

Reviewers' comments:

# Editor report: Acceptance letter

**DOI:** 10.1371/journal.pone.0361590.r006

**Authors:** Anne E. Martin

PONE-D-26-26610R2

PLOS One

Dear Dr. Buckley,

I'm pleased to inform you that your manuscript has been deemed suitable for publication in PLOS One. Congratulations! Your manuscript is now being handed over to our production team.

At this stage, our production department will prepare your paper for publication. This includes ensuring the following:

* All references, tables, and figures are properly cited

* All relevant supporting information is included in the manuscript submission,

* There are no issues that prevent the paper from being properly typeset

You will receive further instructions from the production team, including instructions on how to review your proof when it is ready. Please keep in mind that we are working through a large volume of accepted articles, so please give us a few days to review your paper and let you know the next and final steps.

Lastly, if your institution or institutions have a press office, please let them know about your upcoming paper now to help maximize its impact. If they'll be preparing press materials, please inform our press team within the next 48 hours. Your manuscript will remain under strict press embargo until 2 pm Eastern Time on the date of publication. For more information, please contact onepress@plos.org.

You will receive an invoice from PLOS for your publication fee after your manuscript has reached the completed accept phase. If you receive an email requesting payment before acceptance or for any other service, this may be a phishing scheme. Learn how to identify phishing emails and protect your accounts at https://explore.plos.org/phishing.

If we can help with anything else, please email us at customercare@plos.org.

Thank you for submitting your work to PLOS One and supporting open access.

Kind regards,

PLOS One Editorial Office Staff

on behalf of

Dr. Anne E. Martin

Academic Editor

PLOS One
